# Determinants of the Nucleocytoplasmic Balance of the Cucumber Mosaic Virus and Peanut Stunt Virus 2b Proteins Regulate the Subcellular Localization and RNA Silencing Suppressor Activity

**DOI:** 10.3390/plants15203080

**Published:** 2026-10-09

**Authors:** Dóra Pinczés, Réka Sáray, Attila Fábián, Asztéria Almási, Katalin Salánki

**Affiliations:** 1 Department of Plant Pathology, Plant Protection Institute, Centre for Agricultural Research, Hungarian Research Network (HUN-REN), 1116 Budapest, Hungary; pinczes.dora@atk.hun-ren.hu (D.P.); saray.reka@atk.hun-ren.hu (R.S.); almasi.aszteria@atk.hun-ren.hu (A.A.); 2 Agricultural Institute, Centre for Agricultural Research, Hungarian Research Network (HUN-REN), 2462 Martonvásár, Hungary; attila.fabian@atk.hun-ren.hu

**Keywords:** cucumber mosaic virus, peanut stunt virus, 2b protein, RNA silencing suppressor, nucleocytoplasmic localization, nuclear localization signal, nuclear export signal, viral pathogenicity

## Abstract

The 2b protein of cucumoviruses is an RNA silencing suppressor (RSS), and its activity is associated with its nucleocytoplasmic localization. However, the contribution of nuclear localization signals (NLSs), nuclear export signals (NESs), and their sequence context remains poorly understood. Here, we compare cucumber mosaic virus (CMV) and peanut stunt virus (PSV) 2b proteins and their NLS- and NES-containing regions using recombinant proteins. The subcellular localization of PSV 2b has not been characterized, and no protein structural model has been reported previously. CMV 2b displayed a balanced nucleocytoplasmic distribution and strong RSS activity, whereas PSV 2b showed predominantly nuclear localization and weaker RSS activity. Exchange of the N-terminal region and NLSs resulted in pronounced changes in localization and RSS activity. PPC 2b, containing the CMV C-terminal region preceded by PSV sequence, showed the highest RSS activity, while the corresponding recombinant virus caused the most severe symptoms. AlphaFold analysis demonstrated lower structural confidence and higher predicted flexibility in the NES-containing C-terminal regions of CMV 2b and PPC 2b, compared with PSV 2b and CCP 2b, containing the PSV C-terminal region preceded by CMV sequence. These differences may influence NES accessibility and nuclear export.

## 1. Introduction

The nucleus of a plant cell is a highly regulated compartment separated from the cytoplasm by the nuclear envelope, which serves as a barrier controlling the exchange of macromolecules between these two cellular compartments. The dynamic distribution of proteins between the nucleus and cytoplasm represents an important mechanism for regulating protein activity and cellular functions such as gene expression, cell cycle regulation, or signal transduction [1,2].

Nuclear import is mediated by members of the karyopherin family of proteins. Proteins that can enter the nucleus usually contain nuclear localization signals (NLSs), which are recognized by importin proteins. Characterized NLS motifs are typically enriched in basic amino acids, lysine and arginine residues [1,2,3]. In contrast, nuclear export is commonly mediated by nuclear export signals (NESs), which are frequently characterized by leucine-rich hydrophobic motifs recognized by the export receptor CRM1/XPO1 [4,5]. Although nuclear import mechanisms have been extensively characterized, considerably less is known about nuclear export pathways, particularly in plant systems [6,7].

Plant DNA viruses, such as begomoviruses or caulimoviruses, naturally replicate in the nucleus and therefore directly depend on nuclear processes. In contrast, most positive-sense RNA plant viruses replicate in the cytoplasm, while some of their proteins also target the nucleus, where they hijack host nuclear functions despite their cytoplasmic replication [8,9,10,11]. This intracellular trafficking is mediated by NLSs and NESs of the viral proteins, which are recognized by the host importin and exportin transport machinery. The ability to shuttle between the nucleus and the cytoplasm is a common strategy among plant viruses, allowing viral proteins to coordinate multiple stages of the infection cycle while interacting with host regulatory pathways. Nuclear trafficking has important roles in viral replication, cell-to-cell and long-distance movement, suppression of RNA silencing, modulation of host gene expression, and the establishment of compatible host–virus interactions [7].

Functional NLSs and, in a smaller number of cases, NESs have been identified in proteins encoded by diverse plant virus families. In many cases, disruption of these targeting signals alters subcellular localization and compromises viral infectivity or pathogenicity, demonstrating that nucleocytoplasmic trafficking is required for the optimal functioning of the virus [12,13,14]. The triple gene block protein 1 (TGBp1, movement protein) of hordeiviruses harbors functional nuclear localization signals that facilitate its nuclear transport. Nuclear targeting of TGBp1 has been associated with efficient virus movement, pathogenicity, and interactions with host defense pathways [15]. The movement protein (ORF3) of groundnut rosette virus (GRV) contains an arginine-rich NLS that mediates its nuclear and nucleolar targeting. ORF3 also contains a leucine-rich NES which is important for nuclear export. Both nucleolar trafficking and the interaction with fibrillarin are required for long-distance virus movement. Mutations affecting L149 or L153 of the NES impair nuclear export, thus disrupt the long-distant movement, demonstrating that coordinated nuclear export is critical for ORF3 function [13,16]. A NES signal was also identified in the p25 protein of beet necrotic yellow vein virus (BNYVV), which is involved in the nuclear export of the protein [12]. The C4 protein of tomato leaf curl Yunnan virus (TLCYnV) undergoes XPO1/CRM1-dependent nuclear export. Inhibition of nuclear export or disruption of the NES causes nuclear retention of C4 and impairs its ability to induce developmental abnormalities [17]. The P6 protein of the cauliflower mosaic virus (CaMV) contains a leucine-rich NES within its N-terminal region. Nuclear export of P6 is mediated by the export receptor CRM1/XPO1, and disruption of this NES results in nuclear retention of P6 and compromises its cytoplasmic functions required for efficient CaMV infection, demonstrating the importance of regulated nuclear export for CaMV infection [9]. Recent studies have further demonstrated that regulated nucleocytoplasmic transport is essential for plant virus infection. The NIb replication protein of the turnip mosaic virus (TuMV) contains functional NLS and NES motifs, and XPO1-mediated trafficking between the nucleus and cytoplasm is required for efficient viral replication and systemic infection [18].

The cucumber mosaic virus (CMV, *Cucumovirus CMV*) and peanut stunt virus (PSV, *Cucumovirus PSV*) belong to the genus Cucumovirus and have a similar genome organization, although their host ranges are very different. While CMV has more than 1000 host plants [19], the host range of PSV is restricted mostly to legume species [20,21,22]. The cucumovirus genome comprises three positive-sense single-stranded RNA molecules that encode five proteins: 1a, 2a, 2b, 3a, and the coat protein (CP). Cucumovirus RNA2 encodes the multifunctional 2b protein involved in symptom development [23], cell-to-cell movement [24], and interference with plant salicylic acid- and jasmonic acid-dependent defense pathways [25]. The most important function of the 2b protein is suppressing plant antiviral gene silencing [26]. The 2b protein can interact with both the protein and RNA components of the gene-silencing complex [27,28]. Beyond protein–protein interactions, the 2b protein of CMV binds small interfering RNAs (siRNAs) [29]. This double-stranded RNA (dsRNA) binding domain is located in the N-terminal region (amino acids 1–61) [30].

Functional NLS sequence motifs have been identified on the 2b protein, which contains two arginine-rich sequences located at amino acid residues 22–27 and 33–36. These motifs are recognized by the Arabidopsis importin α (IMPα), which mediates the nuclear and nucleolar import of the 2b protein [30,31]. The NLS regions partially overlap with the small RNA (sRNA)-binding domain [32].

Much less data is available connected to the NESs of the cucumoviral 2b proteins. Based on multiple sequence comparisons, Lucy et al. suggested the possibility of a NES in CMV 2b consisting of leucine- and isoleucine-rich motifs in the N-terminal region of the protein. These motifs showed characteristics of previously described NES sequences and suggested that 2b might undergo nucleocytoplasmic shuttling. However, the functional activity and precise contribution of these putative NES motifs remained unclear [33] until Kim et al. identified and experimentally validated a NES on the 2b protein, a leucine-rich motif in 2b’s C-terminal half [14].

Although CMV genomic RNAs replicate in the cytoplasm, the 2b protein localizes to both the cytoplasm and the nucleus, consistent with the presence of its two NLS motifs [31,34]. Distinct subcellular localization patterns have been reported between the two CMV subgroups. In subgroup I, the 2b protein accumulates in the nucleus and cytoplasm at comparable levels, whereas in subgroup II, it predominantly localizes to the nucleus [34,35].

The subcellular distribution of 2b protein is associated with its biological functions; however, several questions remained open in this respect. Nuclear and nucleolar accumulation has been linked to enhanced symptom development, whereas cytoplasmic localization is essential for efficient suppression of RNA silencing [34,35]. The relationship between nuclear-cytoplasmic distribution and RNA silencing suppressor (RSS) activity is complex. Although early studies suggested an important role for nuclear targeting through NLS motifs [33], the excessive nuclear accumulation may reduce suppressor function [35]. Recent studies have shown that the biological activity of the CMV 2b protein depends on its ability to shuttle between the nucleus and the cytoplasm. Importin α-mediated nuclear import and XPO1-dependent nuclear export are both required for efficient RSS activity, highlighting the importance of balanced nucleocytoplasmic trafficking for 2b function [14].

Intracellular trafficking of PSV 2b has not been validated experimentally; only sequence analyses identified the putative conserved nuclear localization motifs, including both monopartite and bipartite NLSs. These findings suggest that nuclear targeting is a conserved feature among cucumoviral 2b proteins [33].

Increasing evidence indicates that the biological activity of cucumoviral 2b proteins depends on a finely regulated balance between nuclear import and export. However, the mechanism by which different combinations of NLS and NES influence intracellular trafficking and protein function has remained unclear. Therefore, investigating the contribution of different NLS and NES combinations to the intracellular localization of cucumoviral 2b proteins in connection with symptom development and suppressor activity provides new insights into the molecular mechanisms underlying their biological functions.

## 2. Results

### 2.1. Sequence Comparison and Prediction of Nuclear Localization and Export Signals in CMV and PSV 2b Proteins

**Figure 1:**
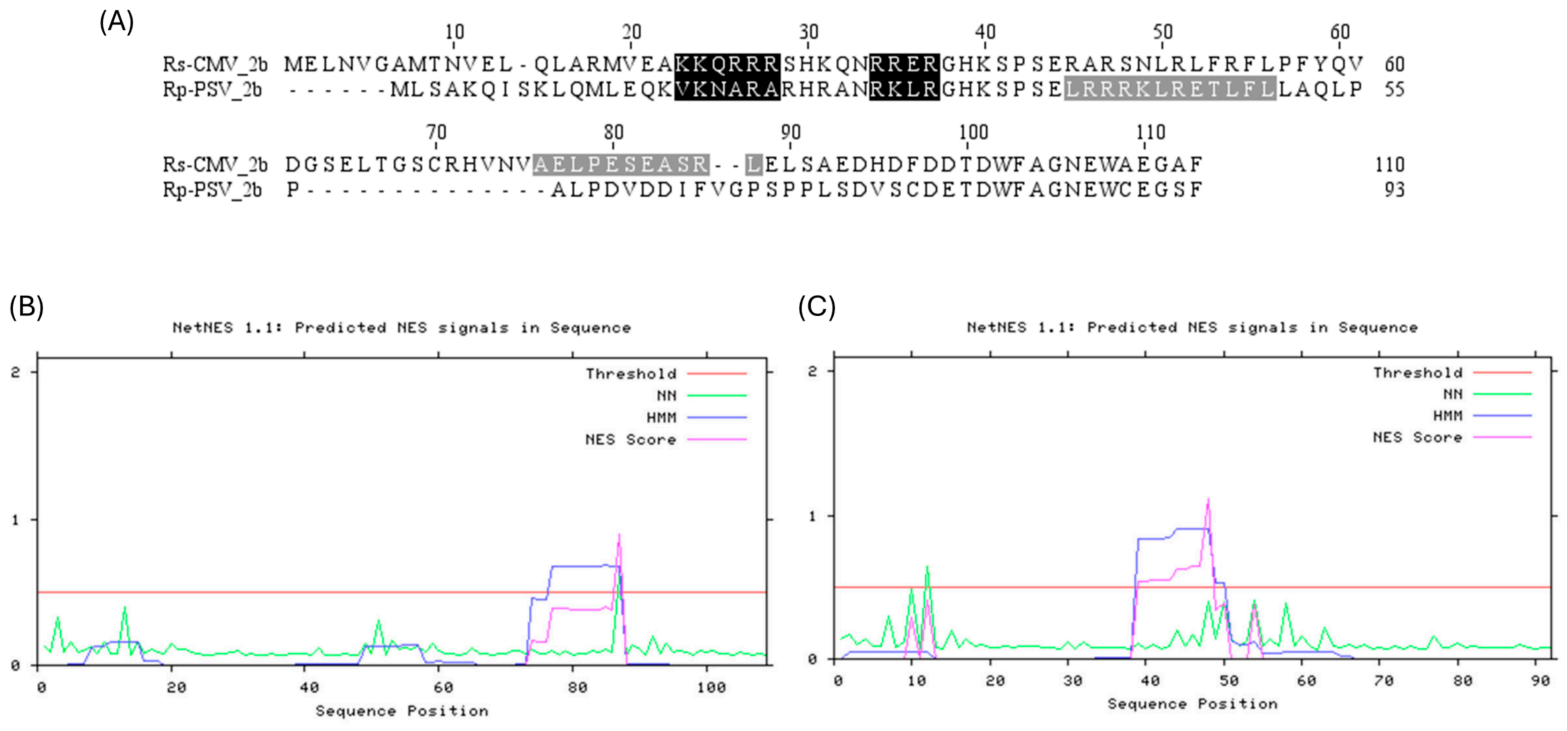
Nuclear localization signals (NLSs) and nuclear export signals (NESs) of Rs-CMV 2b and Rp-PSV 2b. (**A**) CLUSTALW alignment of the Rs-CMV 2b protein and Rp-PSV 2b protein. Black highlights indicate NLSs, and gray highlights indicate NESs. (**B**) NES prediction of the Rs-CMV 2b protein by the NES prediction NetNES 1.1 Server. Amino acid positions above the red threshold line are the predicted NES domains. (**C**) NES prediction of the Rp-PSV 2b protein by the NES prediction NetNES 1.1 Server. Amino acid positions above the red threshold line are the predicted NES domains. NN: Neural network prediction; HMM: hidden Markov model prediction; NES score: the final combined score derived from the NN and HMM predictions; red threshold line: the cut-off value above which a sequence region is predicted as a potential NES.

To investigate the molecular background of the distinct intracellular localization patterns of CMV 2b and PSV 2b proteins, the amino acid sequences of the Rs-CMV and Rp-PSV 2b proteins were compared. Conserved regions corresponding to the previously described NLS1 and NLS2 of CMV 2b were recognized in both 2b protein sequences. The CLUSTALW sequence alignment revealed several major sequence differences throughout the proteins (Figure 1A). The most pronounced differences were found in the N-terminal region preceding NLS1 and around the predicted NLS domains, whereas the C-terminal regions showed a high degree of sequence conservation.

The NLS regions were identified based on the basic residue-rich motifs corresponding to the previously experimentally characterized NLSs of Rs-CMV 2b [31]. In addition, putative NES regions were predicted using the NetNES 1.1 Server. For the CMV 2b protein, the prediction (Figure 1B) was consistent with the previously described experimentally characterized NES region [14]. The corresponding region of Rp-PSV 2b was also analyzed for a potential NES (Figure 1C).

Sequence comparison of the two 2b proteins revealed both conserved and divergent regions, providing a basis for further investigation of sequence differences that may contribute to their distinct nucleocytoplasmic localization. These differences provided the basis for generating recombinant CMV/PSV 2b proteins in which the N-terminal region, the two NLS-containing regions, or the NLS-containing and C-terminal NES-containing regions were exchanged between the two proteins.

### 2.2. Subcellular Localization of CMV, PSV and Recombinant 2b Proteins

**Figure 2:**
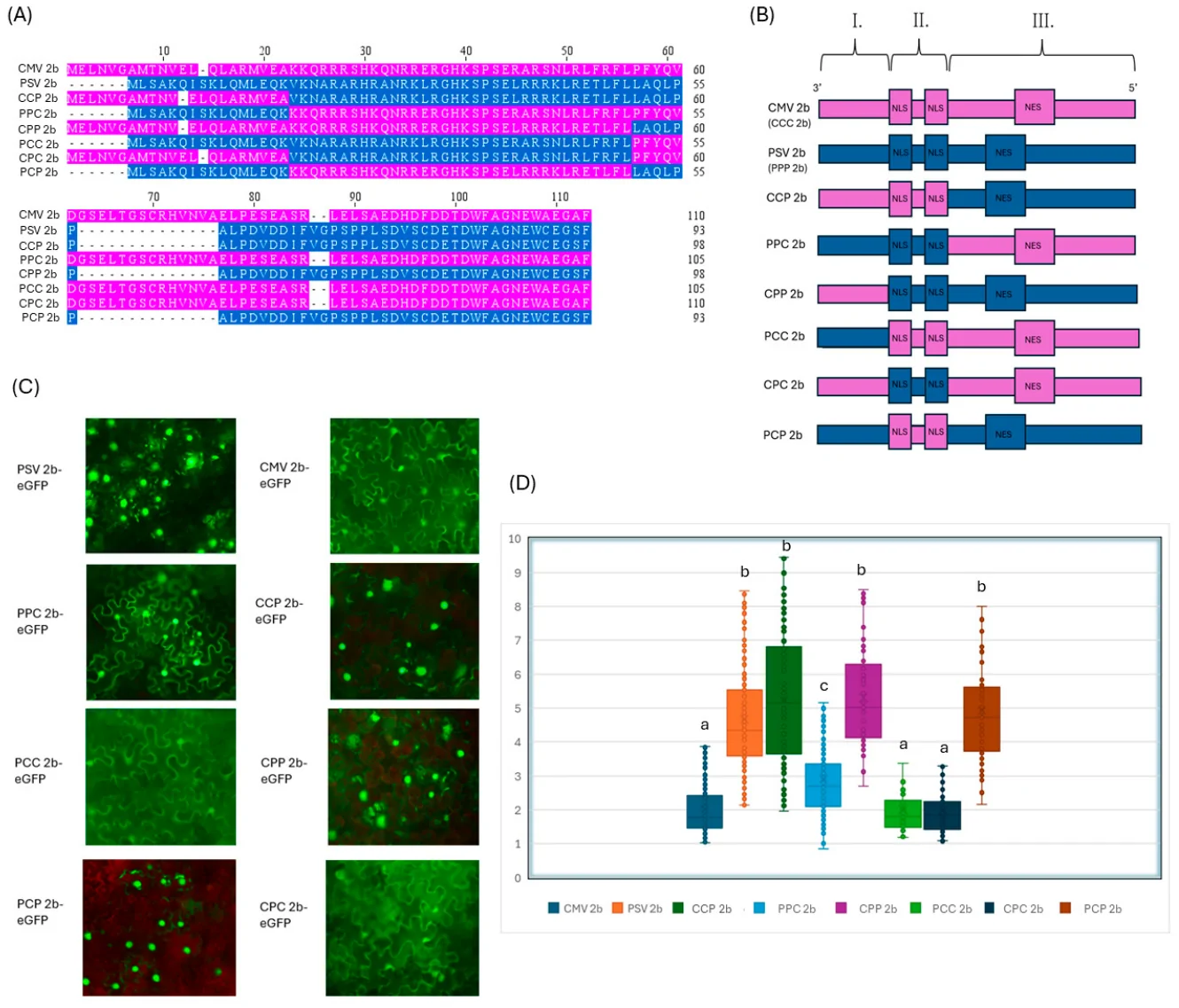
Subcellular localization of CMV 2b, PSV 2b and recombinant 2b-eGFP fusion proteins in *Nicotiana benthamiana* epidermal cells. (**A**) CLUSTALW amino acid sequence alignment of the eight 2b proteins used in this study. Sequences derived from CMV are marked in purple; sequences originating from PSV are marked in blue. (**B**) Schematic representation of the recombinant 2b proteins with highlighted NLS and NES regions. Purple regions originated from CMV; blue regions originated from PSV. The 2b protein was divided into three sequence segments: I, the N-terminal segment preceding the NLSs; II, the segment including the two NLSs; and III, the C-terminal segment following the NLSs, which also contains the NES or predicted NES. The letters C and P indicate the viral origin of each segment, with C referring to CMV and P to PSV. The nomenclature of the recombinant proteins reflects the viral origin of segments I–III, respectively. (**C**) Representative fluorescence microscopy images showing the intracellular distribution of CMV 2b-eGFP, PSV 2b-eGFP, and the recombinant PPC 2b-eGFP, CCP 2b-eGFP, PCC 2b-eGFP, CPP 2b-eGFP, PCP 2b-eGFP, and CPC 2b-eGFP proteins. GFP fluorescence was used to visualize the subcellular localization of the fusion proteins. (**D**) Nucleus-to-cytoplasm (N/C) fluorescence intensity ratios of the eight wild-type and recombinant 2b proteins are represented as a boxplot with a median (middle line) and whiskers from min to max, generated from the mean values of at least 50 measurements. An N/C ratio close to 1 indicates a relatively balanced distribution between the nucleus and cytoplasm, whereas higher N/C ratios indicate increasingly nuclear-enriched localization. ANOVA was carried out to verify the significant differences that were marked with lowercase letters.

The 2b protein amino acid sequences of the Rs-CMV and Rp-PSV isolates exhibit high sequence similarity, containing two N-terminal nuclear localization signals (NLS1 and NLS2) and a putative NES toward the C-terminal region. Despite this similar organization, the two proteins displayed distinct subcellular localization patterns when expressed as eGFP fusion proteins in *Nicotiana benthamiana* epidermal cells. The CMV 2b-eGFP protein showed a relatively balanced distribution between the nucleus and cytoplasm, whereas PSV 2b-eGFP exhibited a more pronounced nuclear localization (Figure 2).

To evaluate the contribution of different localization signal-containing regions to these distinct localization patterns, a series of recombinant 2b proteins were generated, where defined regions were exchanged between the CMV and PSV 2b proteins (Figure 2A,B). In the NT recombinants, the N-terminal region preceding the NLS1 was exchanged (PCC 2b-eGFP, CPP 2b-eGFP), whereas in the NLS recombinants, the N-terminal region containing both NLSs was exchanged together with the N-terminal region (CCP 2b-eGFP, PPC 2b-eGFP). In addition, two “NLS only” recombinants were generated in which only the NLS1–NLS2 region was exchanged while all the other regions originated from the respective wild-type 2b protein (CPC 2b-eGFP, PCP 2b-eGFP). The nomenclature of the recombinant 2b proteins reflects the viral origin of the three sequence segments (I–III), with C referring to CMV and P to PSV. Segment I represents the N-terminal region preceding the NLSs, segment II encompasses the two NLSs and the intervening amino acids, and segment III represents the C-terminal region following the NLSs, including the NES or predicted NES. (Figure 2B). This set of recombinant proteins allowed us to examine how the N-terminal region, the NLSs, and the putative NES contribute individually and in combination to the distinct intracellular localization pattern of CMV and PSV 2b proteins. Replacement of the N-terminal region preceding NLS1 resulted in distinct localization patterns for the two NT recombinants (PCC 2b-eGFP, CPP 2b-eGFP). The localization of these proteins resembled that of the protein from which the longer C-terminal region, including the NLS- and NES-containing regions, originated. The CCP 2b-eGFP protein displayed an intracellular distribution more similar to PSV 2b, whereas PCC 2b-eGFP showed a pattern similar to CMV 2b.

A different pattern was observed when the region containing both NLSs and the N-terminal region were exchanged. The CCP 2b-eGFP protein, containing the two CMV NLSs together with the N-terminal region and the NES of PSV 2b, showed a predominantly nuclear distribution. In contrast, PPC 2b-eGFP, containing the two PSV NLSs together with the N-terminal region and the NES of CMV 2b, showed a more balanced distribution between the nucleus and cytoplasm like the localization of CMV 2b. Notably, the localization pattern of PPC 2b-eGFP appeared more evenly distributed throughout the cell (Figure 2C,D).

Finally, the effect of replacing the two NLS regions alone was evaluated using the CPC 2b-eGFP and PCP 2b-eGFP recombinant proteins. Replacement of the CMV NLS1–NLS2 region with the corresponding PSV sequences did not alter the intracellular distribution of the protein, which remained similar to that of the wild-type CMV 2b-eGFP. Conversely, replacement of the PSV NLS1–NLS2 region with the corresponding CMV sequences also did not substantially affect the intracellular distribution of the protein; it remained similar to that of the wild-type PSV 2b (Figure 2C,D). Thus, exchanging the two NLS regions between CMV 2b and PSV 2b was not sufficient to markedly alter the intracellular distribution of either 2b protein.

To quantify these differences, the nuclear-to-cytoplasmic fluorescence intensity ratio (Fn/Fc) was calculated for individual cells by measuring fluorescence intensity in defined regions of the nucleus and cytoplasm after background correction. The quantitative analysis confirmed the distinct nuclear versus cytoplasmic distribution of the eight proteins (Figure 2D and Figure S1).

These results suggest that the intracellular distribution of the 2b proteins is not determined exclusively by the two NLSs. Instead, the N-terminal region and the C-terminal region containing the putative NES appear to contribute together with the NLSs to the overall nucleocytoplasmic distribution of the proteins.

### 2.3. Subcellular Localization and Homogeneity of CMV 2b, PSV 2b, PPC 2b and CCP 2b

**Figure 3:**
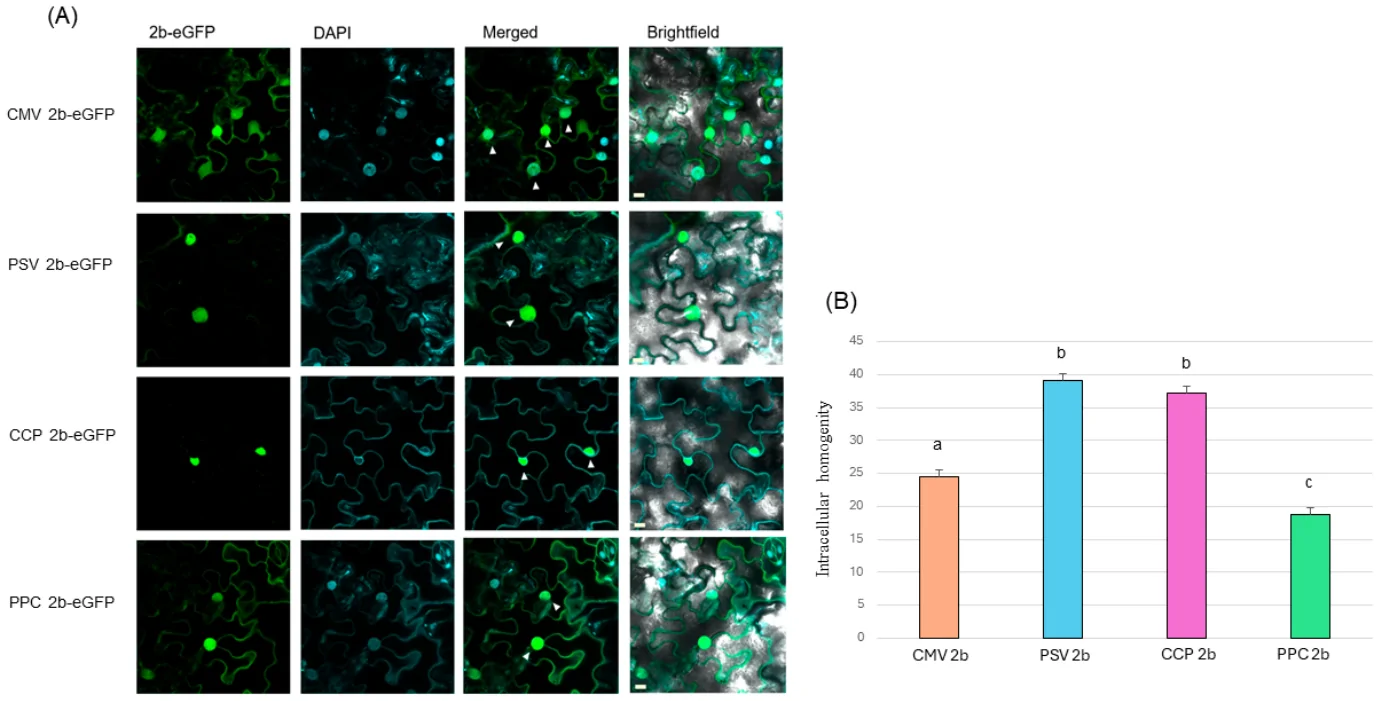
Subcellular localization of eGFP-tagged 2b proteins in epidermal cells of *Nicotiana benthamiana* 24 h post-infiltration, examined in an *Agrobacterium*-mediated transient expression assay. (**A**) Nuclear localization of CMV 2b-eGFP, PSV 2b-eGFP, CCP 2b-eGFP, and PPC 2b-eGFP. The bar represents 10 μm. Cell nuclei were stained with DAPI and marked in cyan; 2b proteins are fused with eGFP, and the green fluorescence was marked in green. Representative nuclei where DAPI signal and GFP signal overlap were indicated with white arrows on merged images. (**B**) Intracellular homogeneity of the 2b proteins in *N. benthamiana* epidermal cells. Significant differences were observed using the Games–Howell multiple comparison test (*p* < 0.05). The different letters above the bars indicate significant differences in fluorescence intensity.

To further characterize the subcellular localization of the wild-type and recombinant 2b proteins, CMV 2b-eGFP, PSV 2b-eGFP, PPC 2b-eGFP and CCP 2b-eGFP were analyzed by confocal laser-scanning microscopy in *N. benthamiana* epidermal cells. Nuclear staining with DAPI was used to distinguish the nuclear and cytoplasmic compartments (Figure 3). Consistent with the observations obtained by conventional fluorescence microscopy, the four proteins displayed distinct intracellular distribution patterns.

CMV 2b-eGFP showed a relatively balanced distribution between the nucleus and cytoplasm, with strong fluorescence detected in both compartments. In contrast, PSV 2b-eGFP showed a more pronounced nuclear accumulation, with a larger proportion of the fluorescence signal localized within the nuclear region. The two recombinant proteins bearing exchanged NLS- and N-terminal regions displayed markedly different localization patterns. CCP 2b-eGFP, which contains the two NLSs derived from CMV 2b and the C-terminal region containing the putative NES from PSV 2b, showed a predominantly nuclear distribution. In contrast, PPC 2b-eGFP, containing the two PSV NLSs and the C-terminal region of CMV 2b, was detected in both the nucleus and cytoplasm and exhibited a distribution closely resembling that of CMV 2b (Figure 3A).

Because the Fn/Fc ratio describes the relative distribution between the nucleus and cytoplasm but does not capture the overall uniformity of the fluorescence signal within the cell, the intracellular homogeneity of the fluorescence distribution was additionally evaluated. Mean values of the intracellular homogeneity were calculated in Figure 3B and Figure S2. Statistically significant differences were observed between the four 2b proteins. Interestingly, PPC 2b-eGFP showed a more homogeneous intracellular fluorescence distribution than CMV 2b-eGFP. Thus, although the PPC 2b-eGFP recombinant exhibited a slightly greater nuclear contribution than the wild-type CMV 2b protein, its fluorescence signal was distributed more homogeneously throughout the cell. For PSV 2b and CCP 2b, fluorescence showed a more uneven distribution between cellular compartments.

The PPC 2b protein exhibited a distinct localization phenotype characterized by a relatively balanced nucleocytoplasmic distribution and increased intracellular homogeneity compared with CMV 2b.

### 2.4. RNA Silencing Suppressor Activity of CMV and PSV 2b Proteins and Recombinant 2b Proteins

Based on the distinct subcellular localization patterns observed among the wild-type and recombinant 2b proteins, four proteins were selected for further functional characterization: CMV 2b, PSV 2b, CCP 2b and PPC 2b. The wild-type CMV 2b and PSV 2b proteins were included as references representing the different localization and RSS activity phenotypes of the two viruses. The two recombinant 2b proteins were selected because they displayed markedly different subcellular distributions based on the combination of the NLS and NES they were harboring.

To investigate whether the exchange of the NLS- and NES-containing regions affects the RSS activity of the 2b proteins, transient co-expression assays were performed in GFP-expressing *N. benthamiana*.

**Figure 4:**
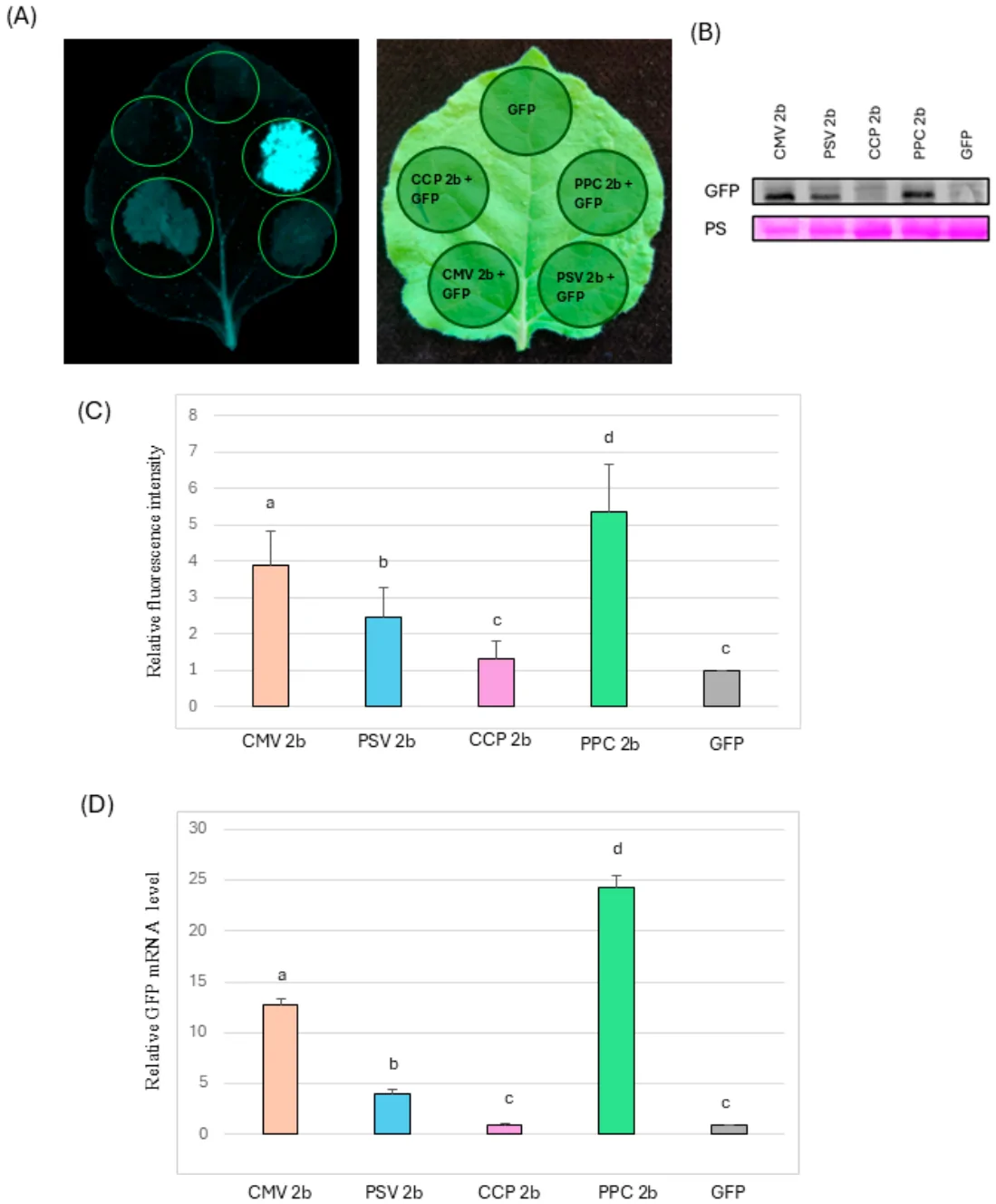
Comparison of the RSS activity of CMV and PSV proteins in patch assays. (**A**) Comparison of the RSS activity of the recombinant 2b proteins used in this study in *Nicotiana benthamiana* and the schematic representation of the infiltrated proteins. (**B**) Detection of GFP protein accumulation in the infiltrated patches by Western blot using an anti-GFP antibody. The GFP control was included as a reference for comparison of GFP protein accumulation. (**C**) Quantification of GFP expression in the infiltrated patches by the measurement of the relative fluorescence intensity. The relative fluorescence was measured using iBright Analysis Software, version 5.6.0. The fluorescence of the degraded GFP-expressing patch collected at 5 dpa was set as 1.0. Significant differences were observed using the Games–Howell multiple comparison test (<0.05). The different lowercase letters above the bars indicate significant differences in fluorescence intensity among the different proteins. (**D**) Relative GFP mRNA levels in the infiltrated patches determined by RT-qPCR. *N. benthamiana* EF1α mRNA was used as the internal reference, and GFP transcript levels were calculated relative to the GFP control, which was set to 1.0. Different lowercase letters indicate significant differences in GFP mRNA levels among the different proteins. Significant differences were observed using the Games–Howell multiple comparison test (*p* < 0.05).

GFP was co-expressed with CMV 2b, PSV 2b, CCP 2b, or PPC 2b, and GFP fluorescence was monitored as an indicator of the suppression of GFP silencing (Figure 4A). As a negative control, GFP was expressed in the absence of a viral suppressor protein. In this control, GFP fluorescence was strongly reduced because of the activation of the plant RNA silencing machinery that induced degradation of GFP mRNA. In contrast, co-expression of the 2b proteins resulted in clearly detectable GFP fluorescence, indicating suppression of GFP silencing.

Marked differences in GFP fluorescence were observed between the 2b proteins. CMV 2b + GFP produced a substantially stronger fluorescence signal than PSV 2b + GFP, consistent with the previously described stronger RSS activity of CMV 2b [29,30]. PSV 2b + GFP also increased GFP fluorescence compared with the GFP-negative control, but the signal was considerably weaker than in the case of CMV 2b. In the two recombinant proteins (CCP 2b and PPC 2b), different effects on GFP mRNA degradation were observed. CCP 2b + GFP produced only a weak fluorescence signal, indicating strongly reduced suppressor activity. In contrast, PPC 2b + GFP resulted in the strongest GFP fluorescence among all tested 2b proteins (Figure 4A).

To quantify the differences observed by fluorescence microscopy, GFP fluorescence was measured in the infiltrated leaf areas (Figure 4C and Figure S3A). The fluorescence values were normalized to the GFP control, which was assigned a relative value of 1. The mean values of relative fluorescence intensities are shown in the bar diagram in Figure 4C. The resulting relative fluorescence intensities and the statistical analyses with the ANOVA model confirmed the visual observations. CMV 2b + GFP showed substantially higher relative fluorescence than PSV 2b, whereas CCP 2b + GFP displayed strongly reduced fluorescence, statistically similar to the negative control GFP. In contrast, PPC 2b + GFP produced the highest relative fluorescence signal of the tested proteins, exceeding the fluorescence level observed for CMV 2b + GFP. Statistical analysis further supported the differences observed between the tested 2b proteins. The relative fluorescence intensities of the infiltrated patches were compared between the groups, and statistically significant differences were detected among the tested proteins.

To further validate the fluorescence-based measurements, GFP mRNA levels were determined by RT-qPCR in the infiltrated leaf patches (Figure 4D). GFP mRNA levels were normalized to the GFP control, which was assigned a relative value of 1. The RT-qPCR results showed a pattern consistent with the fluorescence measurements, with the different 2b constructs producing corresponding differences in GFP mRNA accumulation. Statistical analysis confirmed significant differences among the tested constructs, with the same overall grouping of the treatments as observed for the fluorescence measurements (Figure S3B). Thus, the increased or reduced GFP fluorescence associated with the different 2b proteins was accompanied by corresponding changes in GFP mRNA levels.

In addition, GFP protein accumulation was examined by Western blot using an anti-GFP antibody (Figure 4B). GFP protein was detected in the infiltrated patches, and the observed differences in GFP accumulation were consistent with the relative fluorescence patterns. Although this Western blot was performed as a single analysis and was therefore not used for statistical comparison, it provided additional qualitative evidence at the protein level supporting the fluorescence and RT-qPCR results. The fluorescence measurements, GFP transcript analysis, and additional protein-level detection consistently supported the differences in RSS activity observed among the CMV 2b, PSV 2b, CPP 2b, and PCC 2b proteins.

Taken together, these results demonstrate that exchanging the regions containing the NLSs and the putative NES had a pronounced effect on the RSS activity of the 2b protein. In particular, the recombinants showed opposite functional phenotypes: NLS C/P 2b had no significant RNA silencing suppressor activity as its relative fluorescence intensity did not differ significantly from that of the GFP-only negative control, resulting in the two treatments being assigned to the same statistical group, whereas NLS P/C 2b displayed enhanced activity compared with the wild-type CMV 2b protein. Thus, the region containing the two NLSs combined with the C-terminal region appears to make an important contribution to the functional activity of the 2b protein.

### 2.5. Symptom Development of Recombinant CMV Viruses

**Figure 5:**
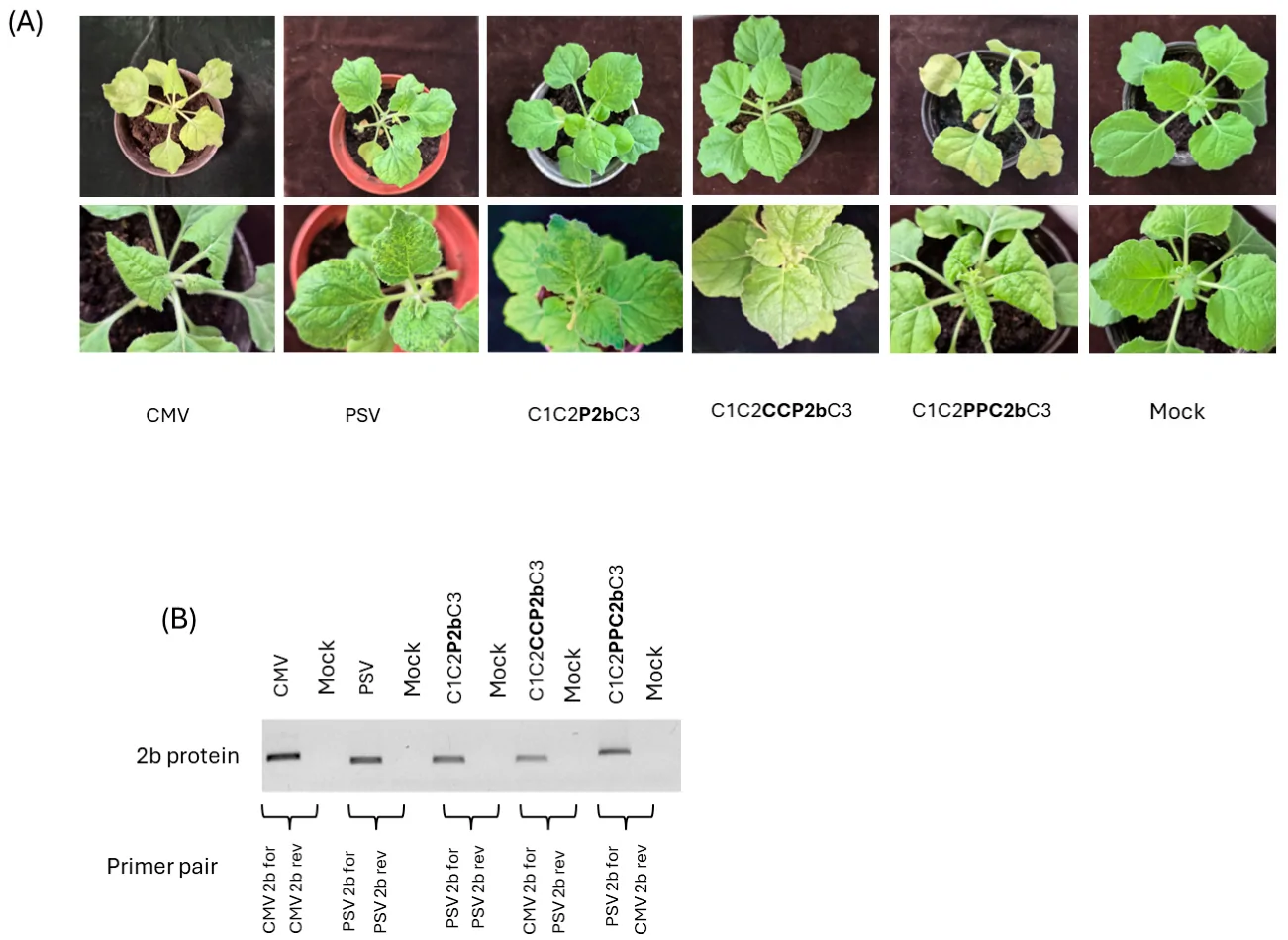
Disease severity induced by CMV viruses carrying wild-type and recombinant 2b proteins in *Nicotiana benthamiana*. (**A**) Disease symptoms observed in *N. benthamiana* plants inoculated with CMV, PSV, and CMV recombinant viruses carrying PSV 2b, CCP 2b, or PPC 2b (C12P2bC3, C12CCP2bC3, C12PPC2bC3) at 14 days post-inoculation (dpi). Bold letters mark the 2b proteins of the virus. C1, C2, and C3 in the virus names indicate the viral origin of RNA1, RNA2, and RNA3, respectively. (**B**) The identity of the recombinant constructs was verified by RT-PCR using primers specific for each 2b-coding region.

To investigate whether the differences recognized in subcellular localization and RSS activity influenced symptom development, infectious CMV clones carrying the recombinant 2b proteins were generated. *N. benthamiana* plants were mechanically inoculated with recombinant viruses containing the wild-type CMV 2b, PSV 2b, CCP 2b, or PPC 2b proteins, and systemic symptom development was monitored two weeks following inoculation (Figure 5). In the nomenclature of the recombinant viruses, C1, C2, and C3 indicate the viral origin of RNA1, RNA2, and RNA3, respectively, with C referring to CMV and P to PSV. The designation following “2b” indicates the viral origin of the corresponding recombinant 2b protein segments.

The wild-type CMV and PSV induced clearly different symptom phenotypes in *N. benthamiana*. CMV induced severe systemic symptoms, whereas infection with PSV resulted in considerably milder symptoms. Replacement of the CMV 2b protein with PSV 2b resulted in attenuation of the symptoms; the 2b recombinant virus (C12P2bC3) produced symptoms like those observed following PSV infection (Figure 5A). Thus, introduction of the PSV 2b into the CMV genome substantially reduced symptom severity.

In contrast, integrating the PPC 2b into the CMV genome (C12PPC2bC3) resulted in the strongest symptom phenotype among the tested viruses. Plants infected with the PPC 2b-containing virus developed even more severe systemic symptoms than those observed in plants infected with wild-type CMV. However, introduction of the CCP 2b protein into the CMV genome (C12CCP2bC3) resulted in a strongly attenuated phenotype, with only very mild symptoms observed in infected plants. Symptoms were even milder than PSV symptoms (Figure 5A). Thus, the two NLS and C-terminal region exchanges produced different effects on virus-induced symptom development.

Interestingly, the symptom severity of the recombinant viruses followed the same trend observed in the RSS assays. The PPC 2b protein, which exhibited the strongest suppressor activity, was associated with the most severe disease phenotype, whereas the CCP 2b protein, which showed strongly reduced suppressor activity, resulted in markedly attenuated symptoms. Similarly, replacement of CMV 2b with the weaker PSV 2b resulted in reduced symptom severity. This analysis was performed to assess the phenotypic effects of the different 2b proteins and their NLS-exchanged variants in the CMV background, rather than to compare viral RNA accumulation.

These findings show that exchanging different regions of the 2b protein can alter the localization and RNA silencing suppressor activity of the recombinant proteins and is associated with changes in the infection phenotype. Together, these results suggest that the sequence context of the NLS- and NES-containing regions may contribute to the functional properties of 2b during viral infection.

### 2.6. Predicted Structural Analysis of CMV and PSV 2b Proteins

**Figure 6:**
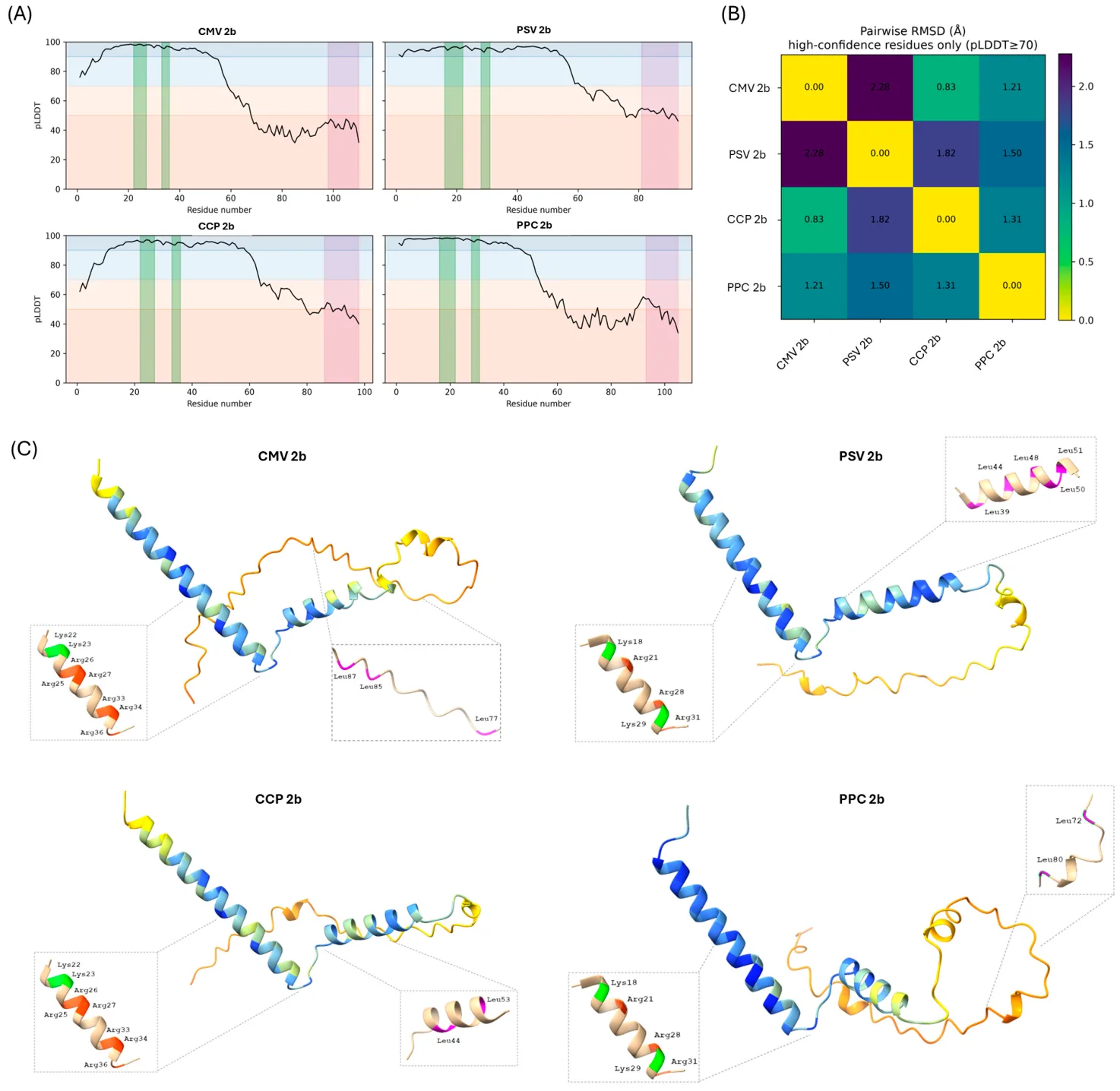
Predicted structural features of CMV 2b and PSV 2b proteins and their recombinant derivatives. (**A**) Per-residue pLDDT confidence for the wild-type and recombinant 2b proteins. The black line represents the predicted local confidence (pLDDT) at each amino acid residue, with values ranging from 0 to 100. The shaded background indicates the standard pLDDT confidence categories, with dark blue representing very high confidence (>90), light blue representing good confidence (70–90), light orange representing low confidence (50–70), and dark orange representing very low confidence (<50). The green vertical regions indicate the reported NLS1 and NLS2 regions. The purple-shaded region indicates the C-terminal region. The N-terminal and NLS-containing regions showed consistently high prediction confidence, whereas confidence generally decreased toward the C-terminal regions. (**B**) Pairwise RMSD comparison of the four predicted 2b structures after restricting the analysis to residues with pLDDT ≥ 70. RMSD values ranged from 0.83 to 2.28 Å, indicating relatively close structural similarity among the confidently predicted regions. (**C**) AlphaFold-predicted structures of Rs-CMV 2b, Rp-PSV 2b, CCP 2b and PPC 2b visualized by ChimeraX 1.12. Structures are colored according to per-residue pLDDT confidence. Blue regions represent higher-confidence predictions whereas yellow and orange indicate lower-confidence regions. The four proteins displayed a broadly related predicted architecture, with the main differences occurring in regions of lower prediction confidence. The NLS and NES regions are shown as enlarged structural views, with arginine (Arg) and lysine (Lys) highlighted in red and green within the NLS region, and leucine (Leu) side chains highlighted in magenta within the predicted NES region.

As no experimentally determined or previously reported three-dimensional structural model of PSV 2b was identified, we generated an AlphaFold model of the Rp-PSV 2b protein and compared it with the corresponding Rs-CMV 2b protein (Figure 6A,C and Figure S4). The predicted PSV 2b structure displayed a broadly similar overall structure to CMV 2b, despite differences in sequence and length. Structural alignment of the two parental proteins resulted in an RMSD of 3.56 Å over 77 aligned residues and a longer-chain-normalized TM-score of 0.50 (Figures S5 and S6). When only residues with pLDDT ≥ 70 were considered, the RMSD decreased to 2.28 Å (Figure 6B), indicating that a substantial proportion of the structural difference between the complete models was associated with lower-confidence regions. Notably, the N-terminal basic regions containing the two NLSs showed very high local confidence, with mean pLDDT values of 96.0–97.9 (Figure 6A). Thus, despite the distinct subcellular localization and RSS activity of the two proteins, their confidently predicted regions retained a broadly related structure.

To evaluate whether the differences in subcellular localization, RSS activity, and pathogenicity observed among the four selected 2b proteins were associated with major changes in their predicted three-dimensional structures, AlphaFold models were generated for Rs-CMV 2b, Rp-PSV 2b, CCP 2b and PPC 2b (Figure 6C). The four proteins displayed broadly related predicted structures, although differences were observed in the complete structural models. Whole-protein structural alignments using TM-align resulted in RMSD values ranging from 2.72 to 3.58 Å, with 66–81 aligned residues per comparison (Figure S5). Longer-chain-normalized TM-scores ranged from 0.50 to 0.62, consistent with broadly related predicted structural topologies (Figure S6).

Notably, restricting the structural comparisons to residues with pLDDT ≥ 70 substantially reduced the pairwise RMSD values, which ranged from 0.83 to 2.28 Å, corresponding to reductions of 36.1–69.4% compared with the complete-model alignments (Figure 6B). The NLS-containing regions showed particularly high local confidence, with mean pLDDT values of 94.6–98.2 across the corresponding regions of the four proteins (Figure 6A). PAE analysis revealed a common pattern among all four models, characterized by low predicted error within the N-terminal region and higher uncertainty within the C-terminal region and in its relative positioning with respect to the N-terminal region (Figure S7). The NES of CMV 2b and PPC 2b is located within a region where the AlphaFold prediction becomes less confident toward the C-terminus, whereas the predicted NES region of PSV 2b and CCP 2b is located within a region that remains more confidently predicted (Figure 6A).

Interestingly, the PPC 2b and CCP 2b proteins, which displayed markedly different subcellular localization, RSS activity, and pathogenicity experimentally, did not exhibit evidence of a unique large-scale structural rearrangement. Their whole-protein RMSD was 3.58 Å, comparable to the 3.56 Å observed between the wild-type CMV and PSV 2b proteins (Figure S5). When only high-confidence residues were considered, the RMSD between CCP 2b and PPC 2b decreased to 1.31 Å over 51 aligned residues (Figure 6B). These results indicate that the pronounced functional differences between the recombinant proteins are not accompanied by major rearrangements of the confidently predicted overall protein fold.

## 3. Discussion

The performance of nuclear localization and export signals is strongly influenced by their sequence context and accessibility within the protein. Post-translational modifications and conformational changes can alter the accessibility of these signals and thereby modulate nucleocytoplasmic trafficking. For example, phosphorylation of the maize Rab17 protein promotes its nuclear accumulation through regulation of NLS activity [36]. Phosphorylation-dependent regulation of intracellular localization has also been demonstrated for CMV 2b [37]. Similarly, phosphorylation can promote nuclear import by disrupting NES function and exposing previously masked NLS motifs, as demonstrated for the extracellular signal-regulated kinase 5 (ERK5), which is a member of the mitogen-activated protein kinase family [38]. These findings indicate that the temporary activity of a nuclear localization signal cannot necessarily be predicted solely from its primary amino acid sequence, but may depend on the surrounding sequence environment and on the balance between import- and export-promoting signals.

This observation is also relevant to the 2b proteins of CMV and PSV. In our study, although both proteins contained highly basic NLS-containing regions in a similar partial protein context, they exhibited markedly different subcellular distributions. CMV 2b protein distribution was relatively balanced between the nucleus and cytoplasm in accordance with previous studies [35,39]. Although previous sequence analyses predicted nuclear localization signals within the PSV 2b protein [33], to our knowledge, the subcellular localization of PSV 2b has not been characterized previously in detail. In our study, Rp-PSV 2b protein displayed predominantly nuclear localization. Thus, the presence of NLSs alone was insufficient to explain the distinct localization patterns of the two proteins. Sequence alignment revealed substantial differences between the N-terminal regions of CMV and PSV 2b proteins, including the regions surrounding the NLSs, while the C-terminal regions were considerably more conserved. In addition, NES prediction identified differences in potential NES between the two proteins. Although our sequence analysis and localization experiments support the presence of a putative NES within residues 39–50 of PSV 2b, its NES activity remains to be experimentally validated by targeted mutagenesis of key residues within this region. These observations suggested that the distinct localization of CMV and PSV 2b may result from the combined activity of multiple nuclear trafficking determinants rather than from the NLSs exclusively.

We further analyzed the sequence determinants underlying the distinct localization patterns of CMV and PSV 2b proteins. To this end, we generated a series of recombinant 2b proteins in which different regions containing NLS and NES or putative NES were exchanged between the two viruses. All in planta experiments presented in this study were conducted using *Nicotiana benthamiana* as the experimental host.

Lucy et al. suggested the presence of a putative NES in the N-terminal region of CMV 2b based on leucine- and isoleucine-rich sequence motifs resembling previously described NES, raising the possibility that this region contributes to nucleocytoplasmic shuttling [33]. However, the functional activity and precise contribution of these motifs remained unclear. Recombinant 2b proteins analyzed in the present study provided further insight into the contribution of this region to 2b localization. Exchange of the N-terminal region preceding the first NLS between CMV and PSV 2b alone did not result in a switch of the overall localization pattern. However, this does not indicate that the N-terminal region is functionally irrelevant. The NLS-only exchange construct provided a complementary perspective: CPC 2b, in which the two NLS regions (NLS1 and NLS2) of CMV 2b were replaced by the corresponding PSV sequences, also showed a localization pattern similar to that of wild-type CMV 2b. Conversely, the reciprocal PCP 2b construct, in which the two NLS regions of PSV 2b were replaced by the corresponding CMV sequences, retained a localization pattern similar to that of wild-type PSV 2b. Thus, neither the N-terminal region preceding the NLSs nor the two NLSs alone was sufficient to change the localization pattern of the 2b protein.

In contrast, when the NLS exchange was extended to include the N-terminal region together with the two NLSs, and the NLSs and NESs or putative NESs originated from different 2b proteins, a pronounced change in localization was observed. The CCP 2b protein, containing the two CMV 2b NLSs together with the PSV-derived C-terminal region containing the putative NES, showed a predominantly nuclear localization. Conversely, the reciprocal PPC 2b protein, containing the PSV NLS region and the CMV-derived C-terminal region with the NES, displayed a more balanced nucleocytoplasmic distribution.

Importantly, quantitative analysis showed that PPC 2b exhibited a more homogeneous intracellular distribution than wild-type CMV 2b. This measurement reflects the uniformity of fluorescence within the cell rather than the relative distribution between the nucleus and cytoplasm. Thus, the more homogeneous distribution of PPC 2b does not contradict the more balanced nuclear–cytoplasmic distribution observed for CMV 2b.

These findings indicate that the N-terminal region and the NLSs do not function as independent localization determinants, but rather contribute to 2b localization in a context-dependent manner. The fact that exchanging the NLSs alone did not alter the localization pattern, whereas their exchange together with the N-terminal region resulted in a pronounced change, suggests that multiple determinants of nucleocytoplasmic trafficking act cooperatively to establish the final intracellular distribution of the protein. This is consistent with previous evidence that CMV 2b undergoes active nucleocytoplasmic shuttling and that its biological activity depends on the coordinated action of nuclear import- and export-promoting elements rather than on NLS alone [14].

Taken together, our results suggest that the NLSs of CMV and PSV 2b are likely to support nuclear import with comparable efficiency, whereas the efficiency of nuclear export may differ between the two proteins. Thus, differences in the NES or in its surrounding sequence context may contribute to the distinct nucleocytoplasmic distribution of CMV and PSV 2b.

These distinct localization patterns raised the question of whether the altered nucleocytoplasmic distribution of the recombinant proteins was also reflected in their biological activity. Since the 2b protein performs its RNA silencing suppressor function in a localization-dependent manner, we also examined whether the different combinations of NLS- and C-terminal regions affected the RSS activity of the recombinant proteins. For further functional analyses, we focused on the CCP and PPC recombinants, as these constructs showed the most pronounced and biologically relevant differences in subcellular localization compared with their respective parental proteins.

The RSS activity assays provided further functional support for our previous findings. Wild-type CMV 2b has strong RNA silencing suppressor activity, whereas PSV 2b exhibited substantially weaker suppressor activity in accordance with previous studies [34,40,41]. The two recombinant proteins containing the NLS region from one virus and the C-terminal region, including the NES or putative NES, from the other showed markedly different activities. CCP 2b exhibited no significant RSS activity, as its fluorescence intensity was statistically indistinguishable from that of the GFP-only negative control. In contrast, PPC 2b displayed even stronger suppressor activity than wild-type CMV 2b. Thus, the stronger nuclear accumulation observed for CCP 2b does not necessarily correlate with increased RSS activity. Instead, excessive nuclear retention could limit the availability of the protein in the cytoplasmic compartment or interfere with the dynamic shuttling required for optimal activity. This interpretation is consistent with the findings of Du et al., who demonstrated that enhanced nuclear and nucleolar accumulation of CMV 2b was associated with reduced RNA silencing suppression [35]. The enhanced RSS activity of PPC 2b may reflect a more effective balance between nuclear import and export, allowing the protein to efficiently shuttle between compartments. This may provide an optimal distribution that enables 2b to perform its functions in both the nucleus and cytoplasm. The importance of this balance is also consistent with the finding of Kim et al. that disruption of the CMV 2b NES reduces the protein’s RSS activity [14].

The biological relevance of these differences was further supported by the inoculation experiments. The recombinant viruses carrying the different 2b proteins in a CMV genomic background displayed disease phenotypes that closely reflected the functional differences observed in the RSS assay. On *N. benthamiana*, Rs CMV induced strong mosaic, dwarfism, and leaf deformation symptoms, while Rp-PSV induced mild mosaic and mild leaf deformation. The replacement of CMV 2b with PSV 2b in CMV resulted in substantially milder disease symptoms, such as in the case of wild-type PSV. Interestingly, the virus carrying PPC 2b induced even stronger symptoms than wild-type CMV, whereas the virus containing CCP 2b produced very mild symptoms. The correlation between RSS activity and disease severity is notable. The PPC 2b protein showed the strongest RSS activity, and the corresponding recombinant virus caused the most severe symptoms. In contrast, CCP 2b displayed limited or no detectable RSS activity, and the corresponding virus caused quite mild symptoms on *N. benthamiana*. These findings further support a close relationship between the RSS activity of 2b and viral pathogenicity. The results also indicate that changes in the 2b protein, particularly in regions involved in its intracellular distribution, can substantially affect viral disease severity. Our observations support a close association between 2b-mediated RNA silencing suppression and viral disease severity. A similar relationship has been observed between CMV subgroups: subgroup I 2b proteins generally show stronger RSS activity and a more balanced nucleocytoplasmic distribution, whereas subgroup II 2b proteins predominantly accumulate in the nucleus and exhibit weaker RSS activity and generally milder symptoms [40,42]. Moreover, the absence of a functional NES in subgroup II 2b proteins has been proposed to contribute to their predominant nuclear accumulation and reduced RSS activity [14]. Thus, the contrasting disease phenotypes of the CMV, PSV, and the recombinant viruses are consistent with previous observations linking 2b nucleocytoplasmic distribution, RSS activity, and viral pathogenicity.

To further investigate whether the differences in subcellular localization, RNA silencing suppressor activity, and pathogenicity observed among the four 2b proteins were associated with changes in their predicted protein structure, AlphaFold models of Rs-CMV 2b, Rp-PSV 2b, CCP 2b, and PPC 2b were generated. To our knowledge, this is the first predicted three-dimensional structural model of PSV 2b and the first structural comparison of PSV 2b with CMV 2b. The four 2b proteins displayed a broadly related predicted structure despite the differences in amino acid sequences, subcellular localization, and biological activity. This result suggests that the functional differences induced by exchanging the NLS- or NES-containing regions of the 2b proteins are not mediated by a major change in the protein structure; rather, they are caused by sequence context, accessibility of targeting motifs, or conformational dynamics.

Interestingly, the experimentally supported NES region of CMV 2b and the corresponding region in PPC 2b are located within the C-terminal region where the AlphaFold prediction becomes progressively less confident, with lower pLDDT values and higher PAE. In contrast, the corresponding predicted NES-containing region of PSV 2b (aa 39–50) and CCP 2b is positioned within a region that remains relatively well predicted. Importantly, the NES activity of the PSV 2b 39–50 region has not been experimentally demonstrated. Therefore, differences in the predicted structural environment of these regions should be interpreted as a potential explanation for differences in nuclear export rather than as evidence that the PSV region functions as an NES. The structural uncertainty surrounding this region could be functionally relevant. The C-terminal region containing the NES may adopt different conformations or exhibit different accessibility depending on the sequence context of the protein. In this context, the different structural environments of the NES regions could potentially influence their accessibility and functional activity. A more dynamic C-terminal region in CMV 2b could allow the NES to become accessible in different conformational states, thereby facilitating efficient nuclear export and nucleocytoplasmic shuttling. In contrast, the putative PSV NES, located within a more confidently predicted region, may be associated with a more constrained structural context and consequently reduced accessibility or activity. Such a difference could contribute to the predominant nuclear localization observed for PSV 2b and, together with other sequence-context-dependent determinants, to its weaker RSS activity. The lower structural confidence of the C-terminal region of the CMV 2b and PPC 2b is also consistent with previous results for cucumoviral 2b proteins. The crystal structure of tomato aspermy virus 2b (TAV 2b) was determined; however, the C-terminal 59–69 residues were not included in the final crystallographic model because of their disordered character [43]. Furthermore, modeling of full-length CMV 2b indicated that its C-terminal domain is partially unstructured [44]. The similar uncertainty for the C-terminal regions of the AlphaFold models in the present study reflects an intrinsic structural property of CMV 2b and PPC 2b proteins rather than a limitation specific to the present predictions. The altered accessibility or activity of the predicted NES of the PSV 2b and CCP 2b could also influence RSS activity, since efficient RSS requires an appropriate balance between nuclear and cytoplasmic localization rather than maximal accumulation in either compartment.

In our study, PPC 2b combined a relatively balanced nucleocytoplasmic distribution and the highest RSS activity among the tested proteins, while the corresponding recombinant virus caused the most severe symptoms. 2b proteins carrying the NES within a less confidently predicted C-terminal region (CMV 2b and PPC 2b) showed a more balanced distribution and stronger RSS activity, whereas those with the NES in a more confidently predicted region (PSV 2b and CCP 2b) were more nuclear and showed weaker RSS activity. Although this does not demonstrate a direct role for structural disorder, it raises the possibility that conformational dynamics of the C-terminal region may influence NES accessibility and nuclear export efficiency, as the intrinsically disordered regions do not adopt a single stable conformation but instead exist as dynamic ensembles of interconverting conformations, which can regulate molecular interactions and protein function [45,46].

At the same time, the differences among the four proteins indicate that the NES-containing and putative NES-containing region alone cannot explain their distinct phenotypes. NLS exchange alone did not alter localization, whereas combined exchange of the N-terminal region and both NLSs markedly affected localization and RSS activity, suggesting that these regions contribute to 2b function in a sequence-dependent context. Overall, our findings support a model in which the coordinated interplay of the N-terminal region, NLSs, and C-terminal region containing experimentally supported or putatively predicted export determinants contributes to the nucleocytoplasmic trafficking, RSS activity, and pathogenicity of 2b proteins. While our results are consistent with a role for the predicted PSV 2b NES-containing region in determining its predominantly nuclear localization, the export activity of this region remains to be experimentally demonstrated.

## 4. Materials and Methods

### 4.1. Plant Material and Growth Conditions

Wild-type *Nicotiana benthamiana* and GFP-expressing transgenic *N. benthamiana* line 16c plants were used for agroinfiltration and inoculation experiments. Plants were cultivated in controlled-environment growth chambers under long-day conditions (16 h light/8 h dark) at 24 °C during the light period and 20 °C during the dark period. All experiments were performed using plants at the 4–5-leaf stage.

### 4.2. Prediction of Nuclear Export Signals

Putative nuclear export signals (NESs) in the CMV and PSV 2b proteins were predicted using the NetNES Server Version 1.1 (Technical University of Denmark, Kongens Lyngby, Denmark). NetNES predicts leucine-rich NES motifs by combining artificial neural network (ANN) and hidden Markov model (HMM) algorithms. The program assigns prediction scores to individual amino acid residues, and regions with scores exceeding the default threshold are identified as candidate NES motifs. For the CMV 2b protein, the predicted NES was evaluated together with the experimentally validated NES previously described by Kim et al. (2022) [14]. The predicted NES regions were subsequently compared between the CMV and PSV 2b proteins and used to support the design and interpretation of recombinant 2b constructs.

### 4.3. Construction of Cucumoviral 2b-eGFP Fusion Proteins and Agroinfiltration Assay for Subcellular Localization

DNA constructs encoding the CMV 2b-eGFP and PSV 2b-eGFP fusion proteins, as well as the recombinant 2b-eGFP variants PPC 2b-eGFP, CCP 2b-eGFP, CCP 2b-eGFP, PCC 2b-eGFP, CPC 2b-eGFP, PCP 2b-eGFP, were generated by overlap PCR. *Sac*I and *Bam*HI restriction sites were introduced into the specific primer pairs; the resulting PCR products were initially cloned into the pGEM-T Easy vector (Promega, Madison, WI, USA); and the nucleotide sequences of the resulting clones were verified by Sanger sequencing (Eurofins Biomi Ltd., Gödöllő, Hungary). The nucleotide sequences of all primers are listed in Table S1. The verified inserts were subcloned into the binary expression vector pBIN61s with the cauliflower mosaic virus (CaMV) 35S promoter and NOS terminator [24]. Finally, the constructs were introduced into *Agrobacterium tumefaciens* strain C58C3.

The construction of the CMV 2b-eGFP fusion protein has been described previously [37].

For transient expression, 4–5-leaf-stage *N. benthamiana* plants were agroinfiltrated with *A. tumefaciens* strains carrying the different 2b-eGFP constructs. To enhance transient protein accumulation, infiltrations were performed together with an *A. tumefaciens* strain harboring the p14 silencing suppressor protein of cymbidium ringspot virus (CymRSV) as described previously [47]. Bacterial suspensions carrying the 2b-eGFP constructs were adjusted to an OD_600_ of 0.2, whereas the p14-expressing strain was adjusted to an OD_600_ of 0.4.

Leaf samples were collected three days after agroinfiltration. GFP fluorescence was first examined by conventional epifluorescence microscopy (Olympus BX51, Olympus Optical Co., Tokyo, Japan). For confocal imaging, leaf tissues were stained with 4′,6-diamidino-2-phenylindole (DAPI) to visualize cell nuclei. Images were acquired using a Leica TCS SP8 confocal microscope (Leica Microsystems GmbH, Wetzlar, Germany) equipped with an HC PL APO CS2 40×/1.10 water immersion objective. GFP was excited at 488 nm, and fluorescence emission was detected between 490 and 540 nm. The confocal aperture was set to 0.652 Airy units. Images were acquired by bidirectional scanning along the x-axis, and three consecutive frames were averaged to reduce image noise. Images were processed using Leica LAS X version 5.1 software (Leica Microsystems GmbH, Wetzlar, Germany).

### 4.4. Quantification of Subcellular Localization and Intracellular Fluorescence Distribution

The subcellular localization of GFP-fused proteins was quantified using ImageJ version 1.54t software (National Institutes of Health, Bethesda, MD, USA). Quantification was as previously described [48,49]. Images acquired by epifluorescence microscopy were used for quantitative analysis, as they more accurately represented the total fluorescence intensity throughout the entire cell than single optical sections obtained by confocal microscopy.

For each cell, the mean fluorescence intensity was measured within a defined 20 × 20 pixel region positioned in the nucleus and also in the cytoplasm. Nuclear regions were selected as close as possible to the center of the nucleus, while representative cytoplasmic regions were chosen to avoid the nuclear envelope and other intracellular structures. Background fluorescence was determined from an area outside the cell and subtracted from both nuclear and cytoplasmic measurements. The comparative images were acquired under consistent imaging conditions, and images containing over-saturated or under-saturated pixels were excluded from quantitative analysis.

The relative nuclear accumulation of each protein was expressed as the nuclear-to-cytoplasmic fluorescence ratio (Fn/Fc), calculated according to the following equation:

[Fn/Fc = (Fn − B)/(Fc − B)]

where Fn and Fc represent the mean fluorescence intensities measured in the nucleus and cytoplasm, respectively, and B represents the mean background fluorescence.

At least 50 cells were analyzed for each construct. The resulting fluorescence ratios were used for statistical analysis.

To evaluate the intracellular distribution of GFP-fused proteins, the homogeneity of the fluorescence signal was quantified using ImageJ version 1.54t software (National Institutes of Health, Bethesda, MD, USA). For each cell, the region of interest (ROI) corresponding to the entire fluorescent cell was manually selected, and the standard deviation (SD) of pixel intensities within the GFP channel was determined.

The standard deviation (SD) of fluorescence intensity was used as a measure of intracellular fluorescence distribution. Lower SD values indicate a more homogeneous distribution of the fluorescent signal throughout the cell, whereas higher SD values reflect a more heterogeneous localization resulting from fluorescence accumulation in specific subcellular compartments, such as the nucleus or nucleolus. At least 50 cells were analyzed for each construct.

### 4.5. Agrobacterium-Mediated RNA Silencing Suppression Assay

For Agrobacterium-mediated transient expression, constructs encoding CMV 2b, PSV 2b, CCP 2b, PPC 2b, and GFP were used. The CMV 2b, PSV 2b, and GFP constructs were cloned using the pBIN61s binary expression vector, as described previously [24,41]. The NLS recombinant 2b clones were amplified from the corresponding plasmids using specific oligonucleotide primer pairs containing *Sac*I and *Bam*HI restriction sites (Table S1). The amplified fragments were first cloned into the pGEM-T Easy vector (Promega, Madison, WI, USA), and the nucleotide sequences of the resulting clones were verified by Sanger sequencing. The verified inserts were subsequently subcloned between the *Sac*I and *Bam*HI restriction sites of the pBIN61s binary expression vector under the control of the CaMV 35S promoter and NOS terminator. The resulting constructs were introduced into *Agrobacterium tumefaciens* strain C58C3 using the freeze–thaw method.

For the RNA silencing suppression assay, two-component transient co-expression was performed by agroinfiltration. *A. tumefaciens* cultures carrying the GFP construct and the respective viral 2b constructs were adjusted to a final OD_600_ of 0.2 and co-infiltrated into the abaxial surface of GFP-expressing transgenic *N. benthamiana* line 16c plant leaves using a 1 mL needleless syringe. GFP infiltrations were used as the control. GFP fluorescence was analyzed five days post-agroinfiltration (dpa) using an iBright FL1500 Imaging System (Thermo Fisher Scientific, Waltham, MA, USA) with an excitation filter of 455–485 nm and an emission filter of 508–557 nm.

For quantitative analysis, fluorescence intensity was measured in at least 10 infiltrated leaf patches for each co-infiltration treatment. The entire infiltrated patch was manually selected as the region of interest (ROI), and the median pixel intensity was determined using iBright Analysis Software version 5.6.0 (Thermo Fisher Scientific, Waltham, MA, USA). Data were obtained from three independent experiments. The fluorescence intensity measured for each 2b-containing treatment was normalized to the corresponding GFP control, which was set to 1. The normalized values were used for statistical analysis and are presented as mean ± standard deviation.

### 4.6. qPCR Analysis of GFP Transcript Levels

To assess GFP transcript levels in the RNA silencing suppressor activity assay, samples were collected from infiltrated areas of *N. benthamiana* 5 days post-infiltration (dpi). Total RNA was extracted using the Promega SV Total RNA Isolation Kit (Promega, Madison, WI, USA) according to the manufacturer’s instructions. One microgram of total RNA was used for cDNA synthesis in a 20 μL reaction using random hexamer primers (Thermo Fisher Scientific, Waltham, MA, USA). Quantitative PCR was performed using SYBR Green Master Mix (Thermo Fisher Scientific) and GFP-specific primer pairs (GFP_for and GFP_rev; Table S1). *N. benthamiana* EF1α mRNA was used as the internal reference, and EF1α was amplified using the ef1_for and ef1_rev primer pair (Table S1) [50]. qPCR was performed using a Bio-Rad CFX96 Touch™ Real-Time PCR Detection System version 3.0 (Bio-Rad Laboratories Inc., Hercules, CA, USA) with the thermal cycling conditions described previously [24]. Relative GFP transcript levels were calculated using the ΔΔCt method [51].

### 4.7. Protein Extraction and Western Blot Analysis

Three leaf disks were collected from agroinfiltrated patches of *Nicotiana benthamiana* leaves and homogenized in 200 μL of 1× protein extraction buffer (4× buffer: 40% glycerol, 240 mM Tris/HCl pH 6.8, 8% SDS, 0.04% bromophenol blue, and 5% β-mercaptoethanol). Protein samples were denatured at 95 °C for 5 min and centrifuged for 1 min at 10,000× *g*. The protein extracts were separated on 12% SDS-PAGE gels, and protein loading was verified by Ponceau staining. GFP was detected by Western blot using a rabbit polyclonal anti-GFP antibody (Agrisera, Vännäs, Sweden), followed by an HRP-conjugated anti-rabbit IgG secondary antibody (Agrisera, Vännäs, Sweden). The signal was visualized using an ECL HRP detection reagent (Thermo Fisher Scientific, Waltham, MA, USA), and chemiluminescence was detected using an iBright FL1500 Imaging System (Thermo Fisher Scientific).

### 4.8. Statistical Analysis

For statistical analysis of the fluorescence intensity measurements and qPCR, data were first analyzed for normality and homogeneity of variances using the Kolmogorov–Smirnov test and Levene’s test, respectively. When more than two groups were compared, one-way analysis of variance (ANOVA) was performed. The null hypothesis (H0) stated that the means of the compared groups were equal, whereas the alternative hypothesis (Ha) stated that at least one group mean differed. A *p*-value < 0.05 was considered statistically significant.

Since ANOVA does not identify which individual groups differ significantly from each other, Tukey’s HSD post hoc test was performed for pairwise comparisons when the assumption of homogeneity of variances was fulfilled. When homogeneity of variances was not assumed, the Games–Howell post hoc test was used. Statistically identical groups were assigned the same uppercase letters.

For the subcellular localization analysis, statistical comparisons were performed on the nuclear-to-cytoplasmic fluorescence intensity ratios (Fn/Fc) and the homogeneity values of the different 2b-eGFP fusion proteins. For the RNA silencing suppression assay, the normalized GFP fluorescence intensities of the different 2b-containing treatments were compared.

All statistical analyses were performed using IBM SPSS Statistics 25. A *p*-value < 0.05 was considered statistically significant.

### 4.9. Construction of Recombinant Infectious Clones and Plant Inoculation

Recombinant CMV infectious clones were generated using the infectious cDNA clones of the Rs-CMV isolate (pC1, pC2 and pC3), which have been described previously [37]. The infectious clones of Rp-PSV isolate (pP1, pP2 and pP3) were also described previously [52].

Recombinant CMV RNA2 clones were constructed by replacing the 2b coding region with the corresponding PSV 2b sequence or with recombinant 2b variants containing exchanged NLS and 5′ regions using overlap PCR with gene-specific primers (Table S1). The resulting overlap PCR products were digested with *Hind*III and *Bam*HI and ligated into the corresponding *Hind*III/*Bam*HI-digested plasmid containing the infectious pRs2 clone. The resulting constructs were designated pCMV2PSV2b, pCMV2CCP2b, and pCMV2PPC2b. These recombinant RNA2 clones were subsequently combined with CMV RNA1 and RNA3 to generate the infectious viruses C1C2PSV2bC3, C1C2CCP2bC3, and C1C2PPC2bC3, respectively.

Infectious RNA transcripts were synthesized in vitro from linearized plasmids using T7 RNA polymerase as described previously [53]. For plant inoculation, RNA1, RNA2, and RNA3 transcripts were combined to generate the following viruses: wild-type CMV (C123), wild-type PSV (P123), CMV carrying the complete PSV 2b gene (C1C2PSV2bC3), CMV containing the recombinant 2b with CMV NLSs and PSV NES (C1C2CCP2bC3), and CMV containing the recombinant 2b with PSV NLSs and CMV NES (C1C2PPC2bC3). Schematic representations of the recombinant viruses are shown in Figure 1B.

*N. benthamiana* plants at the three- to four-leaf stage were mechanically inoculated with in vitro transcripts using carborundum as an abrasive. Symptom development was monitored for up to two weeks after inoculation.

For subsequent biological assays, recombinant viruses were propagated in *N. benthamiana* plants and purified. Purified virions were mechanically inoculated onto experimental plants in 0.03 M sodium phosphate buffer (pH 8.7) containing carborundum.

### 4.10. Virus Detection

Systemic infection of the wild-type and recombinant viruses was confirmed by RT-PCR using virus-specific primers (Table S1). Total RNA was extracted from upper non-inoculated leaves using the SV Total RNA Isolation Kit (Promega, Madison, WI, USA), and first-strand cDNA was synthesized according to the manufacturer’s instructions. Viral accumulation was assessed by PCR amplification of the 2b coding region using specific primer pairs.

### 4.11. AlphaFold-Based Structural Prediction and Comparison of 2b Proteins

The predicted three-dimensional structures of Rs-CMV 2b, Rp-PSV 2b, CCP 2b and PPC 2b were generated using the AlphaFold Server and visualized by ChimeraX 1.12. The amino acid sequences of the four 2b proteins were submitted individually as single-chain protein sequences, and the highest-ranked model based on the AlphaFold Server ranking score was selected for subsequent analysis.

Model confidence was assessed using the predicted local distance difference test (pLDDT) scores and predicted aligned error (PAE) matrices provided by the AlphaFold Server. The AlphaFold-predicted structures were further analyzed using Python packages Biopython version 1.83, NumPy version 1.26, pandas version 2.0, Matplotlib version 3.8, and tmtools version 0.3.0 for structural alignment, RMSD and TM-score calculations, data processing, and visualization. The pLDDT scores were used to assess the confidence of local structural predictions, whereas PAE was used to evaluate the confidence of the predicted relative positioning of different regions within each protein. The predicted structures were structurally aligned pairwise using TM-align implemented through the Python tmtools package. Structural similarity was evaluated using root-mean-square deviation (RMSD), TM-score, the number of structurally aligned residues, and sequence identity within the structural alignment. To assess the similarity of the more reliably predicted structural regions, pairwise structural comparisons were additionally performed after excluding residues with pLDDT values below 70. The resulting structural parameters were used to compare the four 2b protein structures. AlphaFold predictions and structural similarity measures were interpreted as computational structural predictions and were not used as direct evidence of NLS or NES activity, protein disorder, or functional equivalence.

## Data Availability

The original contributions presented in this study are included in the article/Supplementary Material. Further inquiries can be directed to the corresponding author.

## Supplementary Materials

The following supporting information can be downloaded at https://www.mdpi.com/article/10.3390/plants15203080/s1. Figure S1: Statistical analysis of nucleus to cytoplasm (N/C) fluorescence intensity ratios of the eight 2b proteins represented in Figure 2; Figure S2: Statistical analysis of intracellular homogeneity of the 2b proteins in *N. benthamiana* epidermis cells represented on Figure 3; Figure S3: (A) Statistical analysis of the GFP relative fluorescence intensity represented on Figure 4. (B) Statistical analysis of the GFP mRNA represented in Figure 4; Figure S4: Pairwise structural superposition of the predicted 2b proteins; Figure S5: Whole-protein structural comparison based on RMSD; Figure S6: Pairwise structural similarity based on TM-score; Figure S7: Predicted aligned error (PAE) analysis of the four 2b proteins; Table S1: Oligonucleotide primer sequences used in this study.

## Abbreviations

The following abbreviations are used in this manuscript:

AGO	Argonaute
BNYVV	Beet necrotic yellow vein virus
CaMV	Cauliflower mosaic virus
CMV	Cucumber mosaic virus
CP	Coat protein
eGFP	Enhanced green fluorescent protein
GRV	Groundnut rosette virus
NLS	Nuclear localization signal
NES	Nuclear export signal
PAE	Predicted aligned error
pLDDT	Predicted local distance difference test
PSV	Peanut stunt virus
PVX	Potato virus X
RSS	RNA silencing suppressor / RNA silencing suppression
SD	Standard deviation
SiRNA	Small interfering RNA
TAV	Tomato aspermy virus
TLCYnV	Tomato leaf curl Yunnan virus
TuMV	Turnip mosaic virus
XPO1	Exportin 1

## References

[1] Harreman M.T., Kline T.M., Milford H.G., Harben M.B., Hodel A.E., Corbett A.H. (2004). Regulation of Nuclear Import by Phosphorylation Adjacent to Nuclear Localization Signals. J. Biol. Chem..

[2] Nardozzi J.D., Lott K., Cingolani G. (2010). Phosphorylation Meets Nuclear Import: A Review. Cell Commun. Signal.

[3] Lange A., Mills R.E., Lange C.J., Stewart M., Devine S.E., Corbett A.H. (2007). Classical Nuclear Localization Signals: Definition, Function, and Interaction with Importin α. J. Biol. Chem..

[4] Chook Y. (2001). Karyopherins and Nuclear Import. Curr. Opin. Struct. Biol..

[5] Cao L., Fu F., Chen J., Shi H., Zhang X., Liu J., Shi D., Huang Y., Tong D., Feng L. (2022). Nucleocytoplasmic Shuttling of Porcine Parvovirus NS1 Protein Mediated by the CRM1 Nuclear Export Pathway and the Importin α/β Nuclear Import Pathway. J. Virol..

[6] Pemberton L.F., Paschal B.M. (2005). Mechanisms of Receptor-Mediated Nuclear Import and Nuclear Export. Traffic.

[7] Kalinina N.O., Makarova S., Makhotenko A., Love A.J., Taliansky M. (2018). The Multiple Functions of the Nucleolus in Plant Development, Disease and Stress Responses. Front. Plant Sci..

[8] Rojas M.R., Jiang H., Salati R., Xoconostle-Cázares B., Sudarshana M.R., Lucas W.J., Gilbertson R.L. (2001). Functional Analysis of Proteins Involved in Movement of the Monopartite Begomovirus, Tomato Yellow Leaf Curl Virus. Virology.

[9] Haas M., Geldreich A., Bureau M., Dupuis L., Leh V., Vetter G., Kobayashi K., Hohn T., Ryabova L., Yot P. (2005). The Open Reading Frame VI Product of *Cauliflower mosaic Virus* Is a Nucleocytoplasmic Protein: Its N Terminus Mediates Its Nuclear Export and Formation of Electron-Dense Viroplasms. Plant Cell.

[10] Tsai C.-W., Redinbaugh M.G., Willie K.J., Reed S., Goodin M., Hogenhout S.A. (2005). Complete Genome Sequence and In Planta Subcellular Localization of Maize Fine Streak Virus Proteins. J. Virol..

[11] Sharma P., Ikegami M. (2009). Characterization of Signals That Dictate Nuclear/Nucleolar and Cytoplasmic Shuttling of the Capsid Protein of Tomato Leaf Curl Java Virus Associated with DNAβ Satellite. Virus Res..

[12] Vetter G., Hily J.-M., Klein E., Schmidlin L., Haas M., Merkle T., Gilmer D. (2004). Nucleo-Cytoplasmic Shuttling of the Beet Necrotic Yellow Vein Virus RNA-3-Encoded P25 Protein. J. Gen. Virol..

[13] Ryabov E.V., Kim S.H., Taliansky M. (2004). Identification of a Nuclear Localization Signal and Nuclear Export Signal of the Umbraviral Long-Distance RNA Movement Protein. J. Gen. Virol..

[14] Kim H., Shimura H., Sueda K., Masuta C. (2022). Importin/Exportin-Mediated Nucleocytoplasmic Shuttling of Cucumber Mosaic Virus 2b Protein Is Required for 2b’s Efficient Suppression of RNA Silencing. PLoS Pathog..

[15] Semashko M.A., González I., Shaw J., Leonova O.G., Popenko V.I., Taliansky M.E., Canto T., Kalinina N.O. (2012). The Extreme N-Terminal Domain of a Hordeivirus TGB1 Movement Protein Mediates Its Localization to the Nucleolus and Interaction with Fibrillarin. Biochimie.

[16] Kim S.H., MacFarlane S., Kalinina N.O., Rakitina D.V., Ryabov E.V., Gillespie T., Haupt S., Brown J.W.S., Taliansky M. (2007). Interaction of a Plant Virus-Encoded Protein with the Major Nucleolar Protein Fibrillarin Is Required for Systemic Virus Infection. Proc. Natl. Acad. Sci. USA.

[17] Mei Y., Wang Y., Hu T., Yang X., Lozano-Duran R., Sunter G., Zhou X. (2018). Nucleocytoplasmic Shuttling of Geminivirus C4 Protein Mediated by Phosphorylation and Myristoylation Is Critical for Viral Pathogenicity. Mol. Plant.

[18] Zhang M., Gong P., Ge L., Li Y., Chang Z., Qiao R., Zhou X., Wang A., Li F. (2022). Nuclear Exportin 1 (XPO1) Binds to the Nuclear Localization/Export Signal of the Turnip Mosaic Virus NIb to Promote Viral Infection. Front. Microbiol..

[19] Edwardson J.R., Christie R.G. (2018). CRC Handbook of Viruses Infecting Legumes.

[20] Mink G.L., Silbernagel M.J., Saksena K.N. (1969). Host Range, Purification, and Properties of the Western Strain of Peanut Stunt Virus. Phytopathology.

[21] Netsu O., Hiratsuka K., Kuwata S., Hibi T., Ugaki M., Suzuki M. (2008). Peanut Stunt Virus 2b Cistron Plays a Role in Viral Local and Systemic Accumulation and Virulence in Nicotiana Benthamiana. Arch. Virol..

[22] Yamagishi M., Masuta C., Suzuki M., Netsu O. (2015). *Peanut stunt Virus*-Induced Gene Silencing in White Lupin (*Lupinus albus*). Plant Biotechnol..

[23] Lewsey M., Surette M., Robertson F.C., Ziebell H., Choi S.H., Ryu K.H., Canto T., Palukaitis P., Payne T., Walsh J.A. (2009). The Role of the *Cucumber mosaic Virus* 2b Protein in Viral Movement and Symptom Induction. Mol. Plant-Microbe Interact..

[24] Nemes K., Gellért Á., Balázs E., Salánki K. (2014). Alanine Scanning of Cucumber Mosaic Virus (CMV) 2B Protein Identifies Different Positions for Cell-To-Cell Movement and Gene Silencing Suppressor Activity. PLoS ONE.

[25] Lewsey M.G., González I., Kalinina N.O., Palukaitis P., Canto T., Carr J.P. (2010). Symptom Induction and RNA Silencing Suppression by the Cucumber Mosaic Virus 2b Protein. Plant Signal. Behav..

[26] Anandalakshmi R., Pruss G.J., Ge X., Marathe R., Mallory A.C., Smith T.H., Vance V.B. (1998). A Viral Suppressor of Gene Silencing in Plants. Proc. Natl. Acad. Sci. USA.

[27] Zhang X., Yuan Y.-R., Pei Y., Lin S.-S., Tuschl T., Patel D.J., Chua N.-H. (2006). *Cucumber mosaic Virus*-Encoded 2b Suppressor Inhibits *Arabidopsis* Argonaute1 Cleavage Activity to Counter Plant Defense. Genes Dev..

[28] Hamera S., Song X., Su L., Chen X., Fang R. (2012). Cucumber Mosaic Virus Suppressor 2b Binds to AGO4-Related Small RNAs and Impairs AGO4 Activities: CMV2b Binds sRNAs and Impairs AGO4 Activities. Plant J..

[29] Goto K., Kobori T., Kosaka Y., Natsuaki T., Masuta C. (2007). Characterization of Silencing Suppressor 2b of Cucumber Mosaic Virus Based on Examination of Its Small RNA-Binding Abilities. Plant Cell Physiol..

[30] Duan C.-G., Fang Y.-Y., Zhou B.-J., Zhao J.-H., Hou W.-N., Zhu H., Ding S.-W., Guo H.-S. (2012). Suppression of *Arabidopsis* ARGONAUTE1-Mediated Slicing, Transgene-Induced RNA Silencing, and DNA Methylation by Distinct Domains of the *Cucumber mosaic Virus* 2b Protein. Plant Cell.

[31] Wang Y., Tzfira T., Gaba V., Citovsky V., Palukaitis P., Gal-On A. (2004). Functional Analysis of the Cucumber Mosaic Virus 2b Protein: Pathogenicity and Nuclear Localization. J. Gen. Virol..

[32] González I., Martínez L., Rakitina D.V., Lewsey M.G., Atencio F.A., Llave C., Kalinina N.O., Carr J.P., Palukaitis P., Canto T. (2010). Cucumber Mosaic Virus 2b Protein Subcellular Targets and Interactions: Their Significance to RNA Silencing Suppressor Activity. Mol. Plant-Microbe Interact..

[33] Lucy A.P., Guo H., Li W., Ding S. (2000). Suppression of Post-transcriptional Gene Silencing by a Plant Viral Protein Localized in the Nucleus. EMBO J..

[34] González I., Rakitina D., Semashko M., Taliansky M., Praveen S., Palukaitis P., Carr J.P., Kalinina N., Canto T. (2012). RNA Binding Is More Critical to the Suppression of Silencing Function of *Cucumber mosaic Virus* 2b Protein than Nuclear Localization. RNA.

[35] Du Z., Chen A., Chen W., Liao Q., Zhang H., Bao Y., Roossinck M.J., Carr J.P. (2014). Nuclear-Cytoplasmic Partitioning of Cucumber Mosaic Virus Protein 2b Determines the Balance between Its Roles as a Virulence Determinant and an RNA-Silencing Suppressor. J. Virol..

[36] Jensen A.B., Goday A., Figueras M., Jessop A.C., Pagès M. (1998). Phosphorylation Mediates the Nuclear Targeting of the Maize Rab17 Protein. Plant J..

[37] Nemes K., Gellért Á., Almási A., Vági P., Sáray R., Kádár K., Salánki K. (2017). Phosphorylation Regulates the Subcellular Localization of Cucumber Mosaic Virus 2b Protein. Sci. Rep..

[38] Kondoh K., Terasawa K., Morimoto H., Nishida E. (2006). Regulation of Nuclear Translocation of Extracellular Signal-Regulated Kinase 5 by Active Nuclear Import and Export Mechanisms. Mol. Cell. Biol..

[39] Mayers C.N., Palukaitis P., Carr J.P. (2000). Subcellular Distribution Analysis of the Cucumber Mosaic Virus 2b Protein. Microbiology.

[40] Ye J., Qu J., Zhang J.-F., Geng Y.-F., Fang R.-X. (2009). A Critical Domain of the *Cucumber mosaic Virus* 2b Protein for RNA Silencing Suppressor Activity. FEBS Lett..

[41] Pinczés D., Sáray R., Nemes K., Palkovics L., Salánki K. (2024). Viral Coat Proteins Decrease the Gene Silencing Activity of Cognate and Heterologous Viral Suppressors. Sci. Rep..

[42] Gao Y., Yang J., Zhang X., Chen A., Gu Z., Du Z. (2021). The Weak Small RNA-Binding Activity of the 2b Proteins of Subgroup II Cucumber Mosaic Virus Strains Is Insufficient for RNA Silencing Suppression. Front. Microbiol..

[43] Chen H., Yang J., Lin C., Yuan Y.A. (2008). Structural Basis for RNA-silencing Suppression by Tomato Aspermy Virus Protein 2b. EMBO Rep..

[44] Gellért Á., Nemes K., Kádár K., Salánki K., Balázs E. (2012). The C-Terminal Domain of the 2b Protein of Cucumber Mosaic Virus Is Stabilized by Divalent Metal Ion Coordination. J. Mol. Graph. Model..

[45] Holehouse A.S., Kragelund B.B. (2024). The Molecular Basis for Cellular Function of Intrinsically Disordered Protein Regions. Nat. Rev. Mol. Cell Biol..

[46] Mittag T., Kay L.E., Forman-Kay J.D. (2010). Protein Dynamics and Conformational Disorder in Molecular Recognition. J. Mol. Recognit..

[47] Johansen L.K., Carrington J.C. (2001). Silencing on the Spot. Induction and Suppression of RNA Silencing in the *Agrobacterium* -Mediated Transient Expression System. Plant Physiol..

[48] Reina J., Zhou L., Fontes M.R.M., Panté N., Cella N. (2019). Identification of a Putative Nuclear Localization Signal in the Tumor Suppressor Maspin Sheds Light on Its Nuclear Import Regulation. FEBS Open Bio.

[49] Wu W.W., Sun Y.-H.B., Panté N. (2007). Nuclear Import of Influenza A Viral Ribonucleoprotein Complexes Is Mediated by Two Nuclear Localization Sequences on Viral Nucleoprotein. Virol. J..

[50] Shukla A., Hoffmann G., Kushwaha N.K., López-González S., Hofius D., Hafrén A. (2022). Salicylic Acid and the Viral Virulence Factor 2b Regulate the Divergent Roles of Autophagy during Cucumber Mosaic Virus Infection. Autophagy.

[51] Livak K.J., Schmittgen T.D. (2001). Analysis of Relative Gene Expression Data Using Real-Time Quantitative PCR and the 2^−ΔΔCT^ Method. Methods.

[52] Pinczés D., Fábián A., Palkovics L., Salánki K. (2022). Peanut Stunt Virus Movement Protein Is the Limiting Factor in Capsicum Annuum Infection. Virus Res..

[53] Salánki K., Carrère I., Jacquemond M., Balázs E., Tepfer M. (1997). Biological Properties of Pseudorecombinant and Recombinant Strains Created with Cucumber Mosaic Virus and Tomato Aspermy Virus. J. Virol..

