# Supplementary material for: Determinants of the Nucleocytoplasmic Balance of the Cucumber Mosaic Virus and Peanut Stunt Virus 2b Proteins Regulate the Subcellular Localization and RNA Silencing Suppressor Activity
Source: Plants (Basel). 2026 Oct 9;15(20):3080. doi: 10.3390/plants15203080 (PMC13708586; doi:10.3390/plants15203080)
Supplement: Supplementary file 1 [file plants-15-03080-s001.zip › plants-4566612-supplementary.pdf]

Figure S1. Statistical analysis of Nucleus to cytoplasm (N/C) fluorescence intensity ratios of the eight 2b proteins represented in Figure 2

Multiple Comparisons

Dependent Variable: VAR00002

Games-Howell

(I) VAR00001	(J) VAR00001	Mean Difference (I-J)	Std. Error	Sig.	95% Confidence Interval Lower Bound	95% Confidence Interval Upper Bound
CMV 2b	PSV 2b	-2,70179*	,16511	,000	-3,2084	-2,1951
	NLS C/P 2b	-3,39493*	,21089	,000	-4,0454	-2,7444
	NLS P/C 2b	-,88538*	,09465	,000	-1,1740	-,5968
	NT C/P 2b	-3,28650*	,18979	,000	-3,8799	-2,6931
	NT P/C 2b	,01723	,11581	1,000	-,3446	,3791
	CNLSP 2b	,04878	,11519	1,000	-,3078	,4054
	PNLSC 2b	-2,90037*	,18155	,000	-3,4641	-2,3366
PSV 2b	CMV 2b	2,70179*	,16511	,000	2,1951	3,2084
	NLS C/P 2b	-,69314	,25886	,135	-1,4854	,0991
	NLS P/C 2b	1,81641*	,17746	,000	1,2733	2,3595
	NT C/P 2b	-,58471	,24198	,241	-1,3288	,1593
	NT P/C 2b	2,71902*	,18959	,000	2,1379	3,3002
	CNLSP 2b	2,75057*	,18922	,000	2,1713	3,3298
	PNLSC 2b	-,19858	,23557	,990	-,9208	,5237
NLS C/P 2b	CMV 2b	3,39493*	,21089	,000	2,7444	4,0454
	PSV 2b	,69314	,25886	,135	-,0991	1,4854
	NLS P/C 2b	2,50955*	,22070	,000	1,8307	3,1884
	NT C/P 2b	,10843	,27527	1,000	-,7368	,9537
	NT P/C 2b	3,41216*	,23057	,000	2,7034	4,1210
	CNLSP 2b	3,44370*	,23026	,000	2,7362	4,1512
	PNLSC 2b	,49456	,26965	,598	-,3322	1,3213
NLS P/C 2b	CMV 2b	,88538*	,09465	,000	,5968	1,1740
	PSV 2b	-1,81641*	,17746	,000	-2,3595	-1,2733
	NLS C/P 2b	-2,50955*	,22070	,000	-3,1884	-1,8307
	NT C/P 2b	-2,40112*	,20063	,000	-3,0246	-1,7776
	NT P/C 2b	,90261*	,13283	,000	,4924	1,3128
	CNLSP 2b	,93415*	,13229	,000	,5278	1,3405
	PNLSC 2b	-2,01499*	,19286	,000	-2,6109	-1,4191
NT C/P 2b	CMV 2b	3,28650*	,18979	,000	2,6931	3,8799
	PSV 2b	,58471	,24198	,241	-,1593	1,3288
	NLS C/P 2b	-,10843	,27527	1,000	-,9537	,7368
	NLS P/C 2b	2,40112*	,20063	,000	1,7776	3,0246
	NT P/C 2b	3,30373*	,21144	,000	2,6481	3,9594
	CNLSP 2b	3,33527*	,21110	,000	2,6812	3,9894
	PNLSC 2b	,38613	,25349	,794	-,3946	1,1669
NT P/C 2b	CMV 2b	-,01723	,11581	1,000	-,3791	,3446
	PSV 2b	-2,71902*	,18959	,000	-3,3002	-2,1379
	NLS C/P 2b	-3,41216*	,23057	,000	-4,1210	-2,7034
	NLS P/C 2b	-,90261*	,13283	,000	-1,3128	-,4924
	NT C/P 2b	-3,30373*	,21144	,000	-3,9594	-2,6481
	CNLSP 2b	,03155	,14817	1,000	-,4261	,4892
	PNLSC 2b	-2,91760*	,20408	,000	-3,5475	-2,2877
CNLSP 2b	CMV 2b	-,04878	,11519	1,000	-,4054	,3078
	PSV 2b	-2,75057*	,18922	,000	-3,3298	-2,1713
	NLS C/P 2b	-3,44370*	,23026	,000	-4,1512	-2,7362
	NLS P/C 2b	-,93415*	,13229	,000	-1,3405	-,5278
	NT C/P 2b	-3,33527*	,21110	,000	-3,9894	-2,6812
	NT P/C 2b	-,03155	,14817	1,000	-,4892	,4261
	PNLSC 2b	-2,94915*	,20373	,000	-3,5774	-2,3209
PNLSC 2b	CMV 2b	2,90037*	,18155	,000	2,3366	3,4641
	PSV 2b	,19858	,23557	,990	-,5237	,9208
	NLS C/P 2b	-,49456	,26965	,598	-1,3213	,3322
	NLS P/C 2b	2,01499*	,19286	,000	1,4191	2,6109
	NT C/P 2b	-,38613	,25349	,794	-1,1669	,3946
	NT P/C 2b	2,91760*	,20408	,000	2,2877	3,5475
	CNLSP 2b	2,94915*	,20373	,000	2,3209	3,5774

*. The mean difference is significant at the 0.05 level.

ANOVA

VAR00002

	Sum of Squares	df	Mean Square	F	Sig.
Between Groups	1847,009	7	263,858	137,496	,000
Within Groups	1834,584	956	1,919		
Total	3681,592	963			

Test of Homogeneity of Variances

		Levene Statistic	df1	df2	Sig.
VAR00002	Based on Mean	31,172	7	956	,000
	Based on Median	27,098	7	956	,000
	Based on Median and with adjusted df	27,098	7	634,914	,000
	Based on trimmed mean	29,723	7	956	,000

Figure S2. Statistical analysis of Intracellular homogeneity of the 2b proteins in *N. benthamiana* epidermis cells represented on Figure 3

ANOVA

VAR00002

	Sum of Squares	df	Mean Square	F	Sig.
Between Groups	6044,970	3	2014,990	65,205	,000
Within Groups	2781,209	90	30,902		
Total	8826,179	93			

Test of Homogeneity of Variances

		Levene Statistic	df1	df2	Sig.
VAR00002	Based on Mean	2,032	3	90	,115
	Based on Median	1,891	3	90	,137
	Based on Median and with adjusted df	1,891	3	72,893	,139
	Based on trimmed mean	1,958	3	90	,126

Multiple Comparisons

Dependent Variable: VAR00002

	(I) VAR00001	(J) VAR00001	Mean Difference (I-J)	Std. Error	Sig.	95% Confidence Interval Lower Bound	95% Confidence Interval Upper Bound
Tukey HSD	CMV 2b	PSV 2b	-14,61885*	1,76528	,000	-19,2398	-9,9979
		NLS C/P 2b	-12,68576*	1,62040	,000	-16,9275	-8,4441
		NLS P/C 2b	5,78644*	1,41864	,001	2,0729	9,5000
	PSV 2b	CMV 2b	14,61885*	1,76528	,000	9,9979	19,2398
		NLS C/P 2b	1,93309	1,98094	,764	-3,2524	7,1186
		NLS P/C 2b	20,40529*	1,81961	,000	15,6421	25,1684
	NLS C/P 2b	CMV 2b	12,68576*	1,62040	,000	8,4441	16,9275
		PSV 2b	-1,93309	1,98094	,764	-7,1186	3,2524
		NLS P/C 2b	18,47220*	1,67942	,000	14,0760	22,8684
	NLS P/C 2b	CMV 2b	-5,78644*	1,41864	,001	-9,5000	-2,0729
		PSV 2b	-20,40529*	1,81961	,000	-25,1684	-15,6421
		NLS C/P 2b	-18,47220*	1,67942	,000	-22,8684	-14,0760

Figure S3. (A) Statistical analysis of the GFP relative fluorescence intensity represented in Figure 4. (B) Statistical analysis of the GFP mRNA represented in Figure 4.

(A)

ANOVA

VAR00002

	Sum of Squares	df	Mean Square	F	Sig.
Between Groups	235,892	4	58,973	82,506	,000
Within Groups	70,762	99	,715		
Total	306,654	103			

Test of Homogeneity of Variances

		Levene Statistic	df1	df2	Sig.
VAR00002	Based on Mean	12,206	4	99	,000
	Based on Median	11,364	4	99	,000
	Based on Median and with adjusted df	11,364	4	51,919	,000
	Based on trimmed mean	12,023	4	99	,000

Multiple Comparisons

Dependent Variable: VAR00002

Games-Howell

(I) VAR00001	(J) VAR00001	Mean Difference (I-J)	Std. Error	Sig.	95% Confidence Interval Lower Bound	95% Confidence Interval Upper Bound
CMV 2b	PSV 2b	1,44979*	,24139	,000	,7666	2,1330
	GFP	2,89760*	,18203	,000	2,3630	3,4322
	NLSC/P 2b	2,56478*	,22250	,000	1,9277	3,2018
	NLSP/C 2b	-1,44035*	,39026	,009	-2,5923	-,2884
PSV 2b	CMV 2b	-1,44979*	,24139	,000	-2,1330	-,7666
	GFP	1,44781*	,15854	,000	,9835	1,9121
	NLSC/P 2b	1,11499*	,20373	,000	,5320	1,6980
	NLSP/C 2b	-2,89015*	,37987	,000	-4,0197	-1,7606
GFP	CMV 2b	-2,89760*	,18203	,000	-3,4322	-2,3630
	PSV 2b	-1,44781*	,15854	,000	-1,9121	-,9835
	NLSC/P 2b	-,33281	,12796	,128	-,7357	,0701
	NLSP/C 2b	-4,33795*	,34521	,000	-5,4039	-3,2720
NLSC/P 2b	CMV 2b	-2,56478*	,22250	,000	-3,2018	-1,9277
	PSV 2b	-1,11499*	,20373	,000	-1,6980	-,5320
	GFP	,33281	,12796	,128	-,0701	,7357
	NLSP/C 2b	-4,00514*	,36816	,000	-5,1123	-2,8979
NLSP/C 2b	CMV 2b	1,44035*	,39026	,009	,2884	2,5923
	PSV 2b	2,89015*	,37987	,000	1,7606	4,0197
	GFP	4,33795*	,34521	,000	3,2720	5,4039
	NLSC/P 2b	4,00514*	,36816	,000	2,8979	5,1123

*. The mean difference is significant at the 0.05 level.

(B)

ANOVA

VAR00002

	Sum of Squares	df	Mean Square	F	Sig.
Between Groups	2392,935	4	598,234	1310,148	,000
Within Groups	11,415	25	,457		
Total	2404,350	29			

Test of Homogeneity of Variances

		Levene Statistic	df1	df2	Sig.
VAR00002	Based on Mean	11,019	4	25	,000
	Based on Median	9,514	4	25	,000
	Based on Median and with adjusted df	9,514	4	10,323	,002
	Based on trimmed mean	11,001	4	25	,000

Multiple Comparisons

Dependent Variable: VAR00002

Games-Howell

(I) VAR00001	(J) VAR00001	Mean Difference (I-J)	Std. Error	Sig.	95% Confidence Interval Lower Bound	95% Confidence Interval Upper Bound
CMV 2b	PSV 2b	8,75000*	,28604	,000	7,7907	9,7093
	CCP 2b	11,86500*	,23798	,000	10,9479	12,7821
	PPC 2b	-11,43000*	,59110	,000	-13,5665	-9,2935
	GFP	11,77833*	,23167	,000	10,8490	12,7077
PSV 2b	CMV 2b	-8,75000*	,28604	,000	-9,7093	-7,7907
	CCP 2b	3,11500*	,17639	,000	2,4548	3,7752
	PPC 2b	-20,18000*	,56910	,000	-22,3216	-18,0384
	GFP	3,02833*	,16778	,000	2,3553	3,7014
CCP 2b	CMV 2b	-11,86500*	,23798	,000	-12,7821	-10,9479
	PSV 2b	-3,11500*	,17639	,000	-3,7752	-2,4548
	PPC 2b	-23,29500*	,54653	,000	-25,4700	-21,1200
	GFP	-,08667	,05445	,557	-,3051	,1317
PPC 2b	CMV 2b	11,43000*	,59110	,000	9,2935	13,5665
	PSV 2b	20,18000*	,56910	,000	18,0384	22,3216
	CCP 2b	23,29500*	,54653	,000	21,1200	25,4700
	GFP	23,20833*	,54381	,000	21,0268	25,3898
GFP	CMV 2b	-11,77833*	,23167	,000	-12,7077	-10,8490
	PSV 2b	-3,02833*	,16778	,000	-3,7014	-2,3553
	CCP 2b	,08667	,05445	,557	-,1317	,3051
	PPC 2b	-23,20833*	,54381	,000	-25,3898	-21,0268

*. The mean difference is significant at the 0.05 level.

Figure S4. Pairwise structural superposition of the predicted 2b proteins. Pairwise structural alignments of the predicted CMV 2b, PSV 2b, CCP 2b and PPC 2b structures generated using TM-align. Overlapping regions indicate similar predicted backbone geometry, whereas separated regions indicate structural differences or regions not included in the alignment.

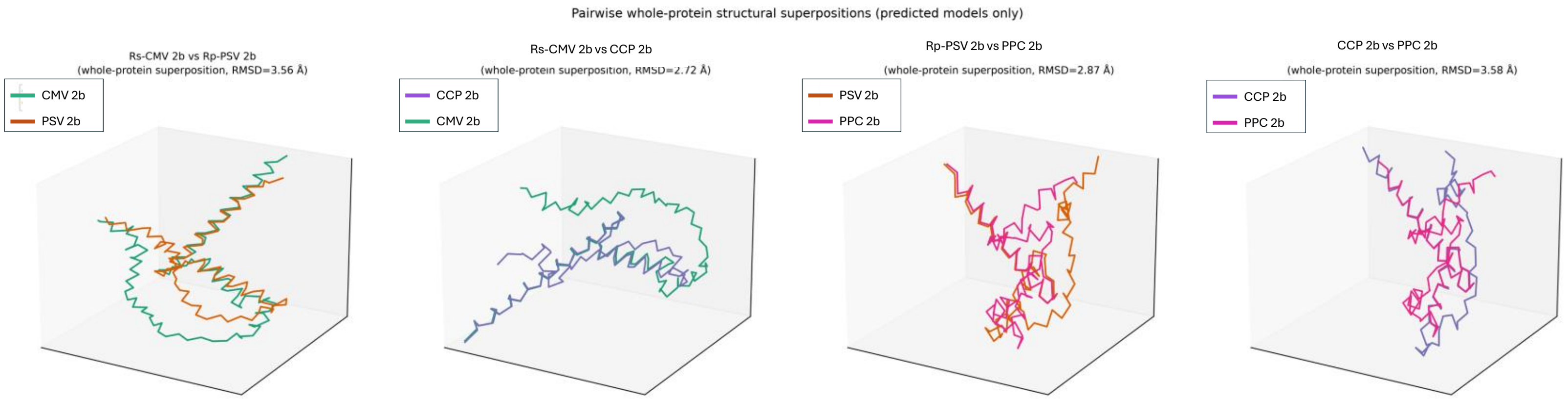

Figure S5. Whole-protein structural comparison based on RMSD. Pairwise RMSD values obtained from TM-align structural superposition of the four predicted 2b protein structures. Lower RMSD values indicate greater structural similarity among the aligned Cα atoms. Diagonal values represent self-comparisons.

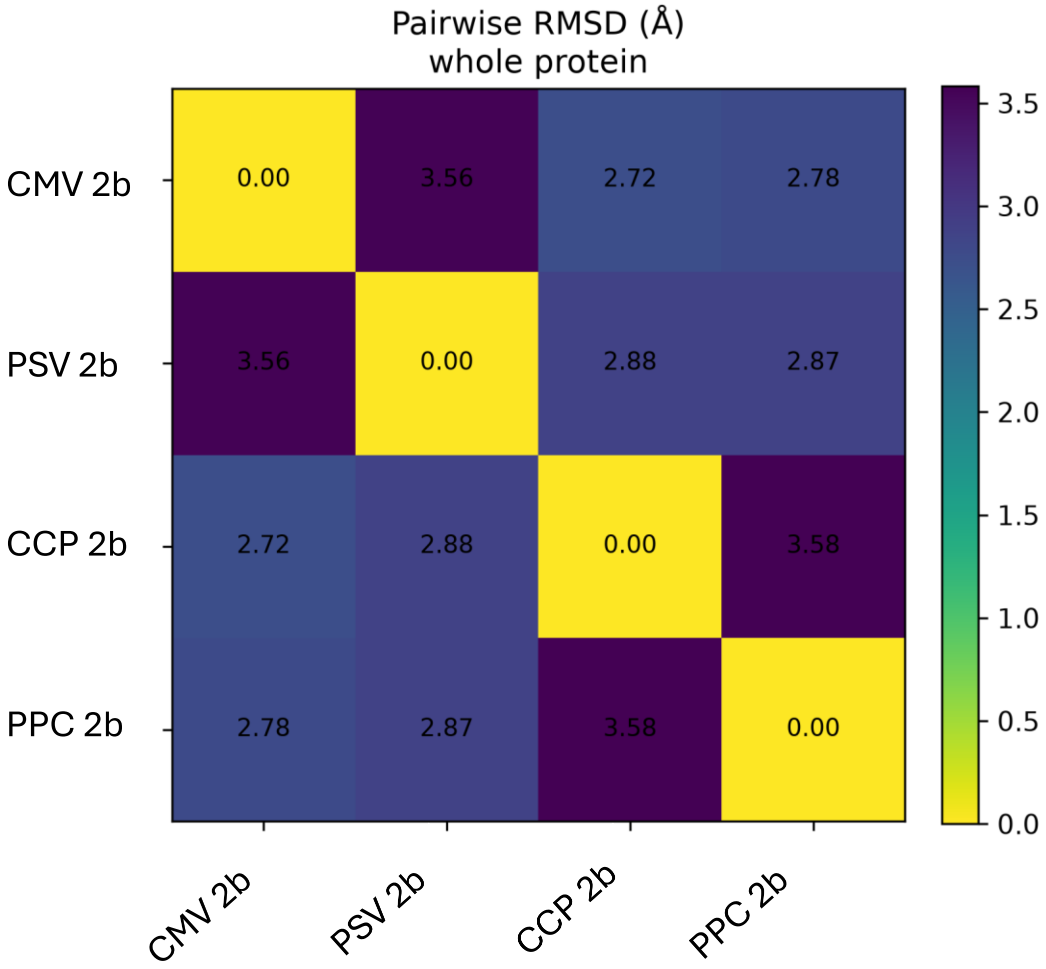

Figure S6. Pairwise structural similarity based on TM-score. Heatmap showing TM-scores obtained from pairwise structural alignment of the four predicted 2b proteins. Values were normalized by the length of the longer protein in each pair. Higher values indicate greater overall structural similarity.

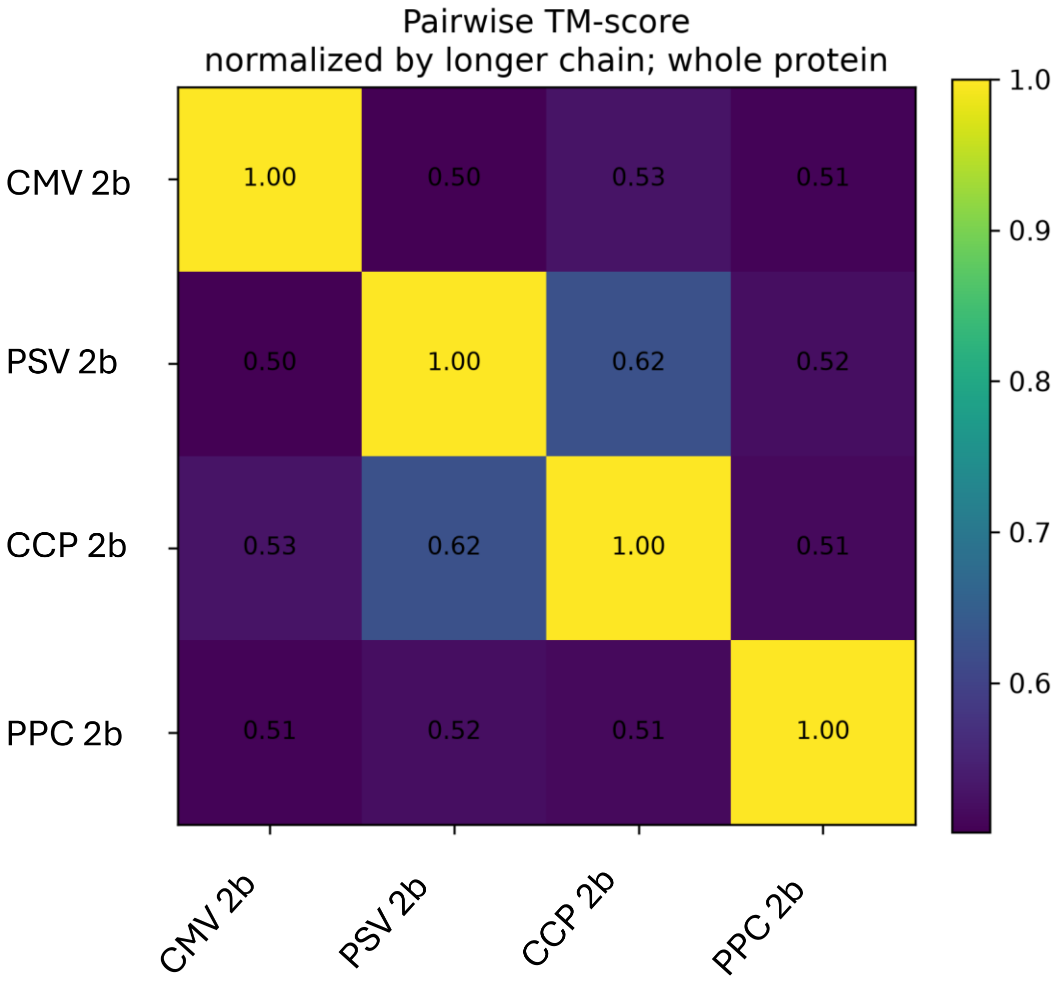

Figure S7. Predicted aligned error (PAE) analysis of the four 2b proteins. Individual PAE matrices for Rs-CMV 2b, Rp-PSV 2b, CCP 2b and PPC 2b, respectively. Darker regions indicate lower predicted aligned error and greater confidence in the relative positioning of residues, whereas lighter regions indicate greater uncertainty.

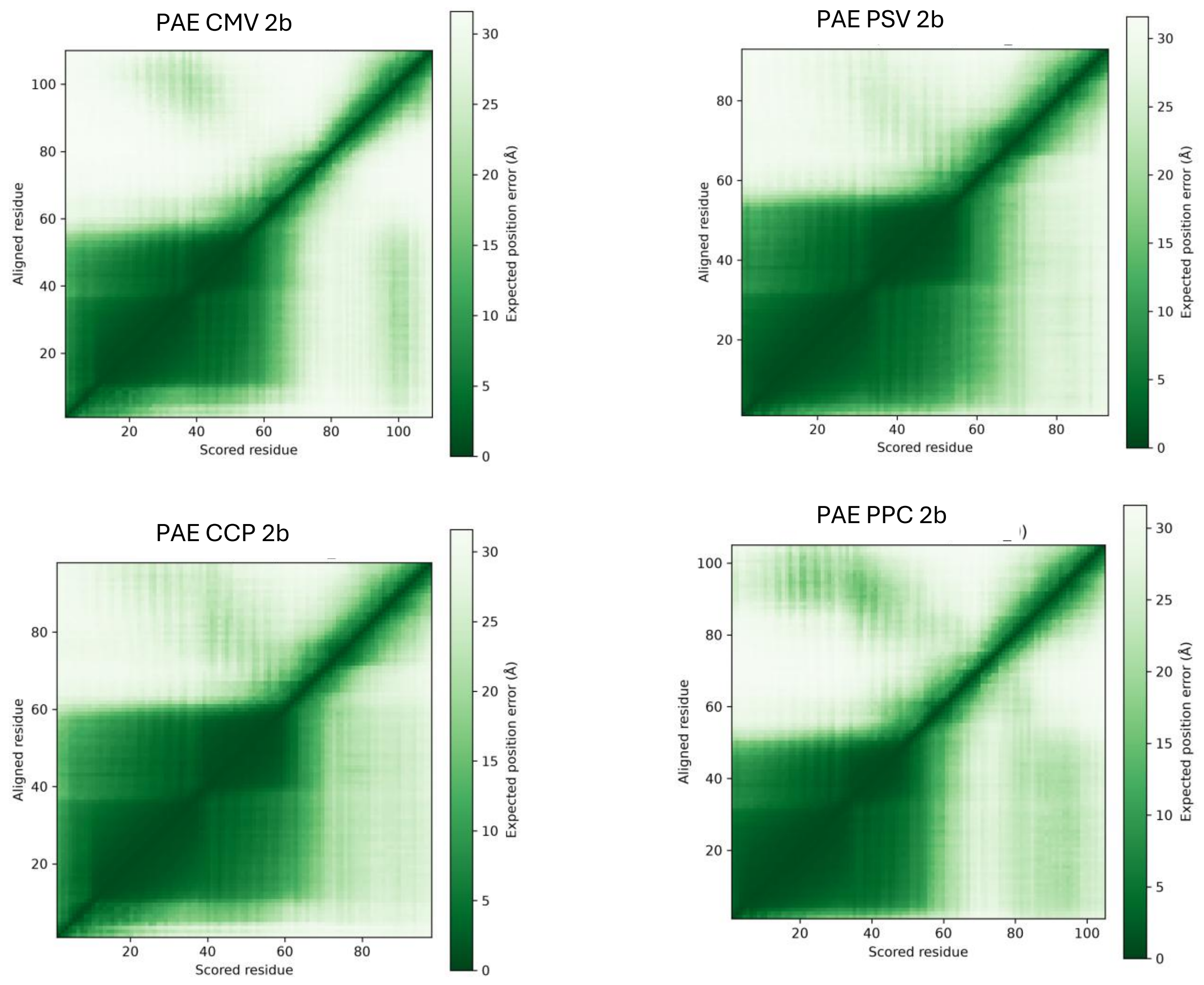

Table S1. Oligonucleotide primer sequences used in this study

Oligonucleotide name	Oligonucleotide sequence	Used for
PSV2bSacI	5'-GGG AGC TCA TGT TGA GTG CAA AGC AGA TCT CAA AA-3'	generating PPC 2b-eGFP, PPC 2b
CMV2bSacIfor	5’-ATT GAG CTC GTA GTA CAG AGTTCA GGG-3’	generating CCP 2b-eGFP, CCP 2b
CMV/PSV 2b 40AAfor	5’-TCG GGA ACG AGG TCA CAA AAG TCC GAG CGA ATT GAG GCG TCG-3’	generating CCP 2b-eGFP, CCP 2b, CPC 2b-eGFP, pCMV2CCP2b
CMV/PSV2b 40AArev	5’-CGA CGC CTC AAT TCG CTC GGA CTT TTG TGA CCT CGT TCC CGT CGA-3’	generating CCP 2b-eGFP, CCP 2b, pCMV2CCP2b
PSV/CMV2b 40AAfor	5’-CCG AAA GTT ACG CGG ACA CAA GAG TCC CAG CGA GAG AGC GCG TTC-3’	generating PPC 2b-eGFP, PPC 2b, PCP 2b-eGFP, pCMV2PPC2b
PSV/CMV2b 40AArev	5’-GAA CGC GTC CTC TCG CTG GAA CTC TTG TGT CCG CGT AAC TTT CGG-3’	generating PPC 2b-eGFP, PPC 2b, PCP 2b-eGFP, pCMV2PPC2b
eGFPBamHIrev	5’-CCG GAT CCT CAC TTG TAC AGC TCG TCC ATG-3’	generating all six recombinant 2b eGFP fusion proteins (CCP 2b-eGFP, CPP 2b-eGFP, CPC 2b-eGFP, PPC 2b-eGFP, PCC 2b-eGFP, PCP 2b-eGFP)
Rs2 -2319for	5'-CTC TAT TCA GAT CGT CGT CAG-3'	Generating pCMV2PPC2b, pCMV2CCP2b
CMV/PSV 21 AA FOR	5'-GGC TCG TAT GGT GGA GGC GAA GGT GAA AAA CGC AAG GGC TCG-3'	Generating CPP 2b-eGFP, PCP 2b-eGFP
CMV/PSV 21 AA REV	5'-CGA GCC CTT GCG TTT TTC ACC TTC GCC TCC ACC ATA CGA GCC-3'	Generating CPP 2b-eGFP, CPC 2b-eGFP
PSV/CMV 21 AA FOR	5'- CCA AAT GTT GGA GCA GAA GAA GCA GAG ACG AAG GTC TC-3'	Generating PCC 2b-eGFP, CPC 2b-eGFP
PSV/CMV 21 AA REV	5'-GAG ACC TTC GTC TCT GCT TCT TCT GCT CCA ACA TTT GG-3'	Generating PCC 2b-eGFP, PCP 2b-eGFP
CMV 2b forw 436	5'-AGA AAT ATG GAA TTG AAC CTA GGT GCA ATG ACA AAC GT-3'	Detecting CMV 2b , CCP 2b
CMV 2bSTOP	5'-GGA TCC TAT AGC TGC CAA CTT AGA GAG AAA GGA TAG TCC TCA GAA AGC ACC TTC CGC CCA TTC -3'	Detecting CMV 2b , PPC 2b, generating PPC 2b
Rp2bSacIFfor	5'-GGA GCT CAA CGT AGG TGC AAT GTT GAG TGC AAA G-3'	Detecting PSV 2b, PPC 2b
PSV 2b Pst rev	5' - GGA TCC TCA GAA ACT ACC CTC ACA CCA CTC A-3'	Detecting PSV 2b, PPC 2b, generating CCP 2b
CMV2017	5'-CAA GAA GAT ACC GTA CTC GGA C-3'	Generating pCMV2PPC2b, pCMV2CCP2b
CMVI.sg.	5’-GCG GAT CCT GGT CTC CTT TTG GAG GCC C-3’	Generating pCMV2PPC2b, pCMV2CCP2b
GFPforward	5’-GTG GAG AGG GTG AAG GTG ATG-3’	RT-qPCR detection
GFPreverse	5’-TGA TCT GGG TAT CTT GAA AAG C-3’	RT-qPCR detection
EF1U	5’-TGG TGT CCT CAA GCC TGG TAT GGT TG-3’	RT-qPCR detection
EF1D	5’-ACGCTTGAGATCCTTAACCGCAACATTCTT-3’	RT-qPCR detection
